# Xylogenesis in zinnia (*Zinnia elegans*) cell cultures: unravelling the regulatory steps in a complex developmental programmed cell death event

**DOI:** 10.1007/s00425-017-2656-1

**Published:** 2017-02-13

**Authors:** Elena T. Iakimova, Ernst J. Woltering

**Affiliations:** 1 Institute of Ornamental Plants, 1222 Negovan, Sofia, Bulgaria; 2 Wageningen University and Research, Food and Biobased Research, P.O. Box 17, 6700 AA Wageningen, The Netherlands; 3 Wageningen University, Horticulture and Product Physiology, P.O. Box 630, 6700 AP Wageningen, The Netherlands

**Keywords:** *Zinnia elegans*, Cell culture, Xylogenesis, Programmed cell death, Signalling, Experimental approaches

## Abstract

**Physiological and molecular studies support the view that xylogenesis can largely be determined as a specific form of vacuolar programmed cell death (PCD). The studies in xylogenic zinnia cell culture have led to many breakthroughs in xylogenesis research and provided a background for investigations in other experimental models in vitro and** ***in planta*** **. This review discusses the most essential earlier and recent findings on the regulation of xylem elements differentiation and PCD in zinnia and other xylogenic systems.**

Xylogenesis (the formation of water conducting vascular tissue) is a paradigm of plant developmental PCD. The xylem vessels are composed of fused tracheary elements (TEs)—dead, hollow cells with patterned lignified secondary cell walls. They result from the differentiation of the procambium and cambium cells and undergo cell death to become functional *post*-*mortem*. The TE differentiation proceeds through a well-coordinated sequence of events in which differentiation and the programmed cellular demise are intimately connected. For years a classical experimental model for studies on xylogenesis was the xylogenic zinnia (*Zinnia elegans*) cell culture derived from leaf mesophyll cells that, upon induction by cytokinin and auxin, transdifferentiate into TEs. This cell system has been proven very efficient for investigations on the regulatory components of xylem differentiation which has led to many discoveries on the mechanisms of xylogenesis. The knowledge gained from this system has potentiated studies in other xylogenic cultures in vitro and *in planta*. The present review summarises the previous and latest findings on the hormonal and biochemical signalling, metabolic pathways and molecular and gene determinants underlying the regulation of xylem vessels differentiation in zinnia cell culture. Highlighted are breakthroughs achieved through the use of xylogenic systems from other species and newly introduced tools and analytical approaches to study the processes. The mutual dependence between PCD signalling and the differentiation cascade in the program of TE development is discussed.

**Electronic supplementary material:**

The online version of this article (doi:10.1007/s00425-017-2656-1) contains supplementary material, which is available to authorized users.

## Introduction

The water-conducting vascular system (xylem) of plants performs two major functions: it provides long-distance water continuum from the soil through the stems, branches and leaves and supports the mechanical strength of these plant organs. In evolutionary aspect, xylem tissue has evolved in terrestrial plants in the process of their adaptation to land habitats (Kenrick and Crane 1997; Friedman and Cook 2000; Tyree 2003; Brodribb 2009; Ligrone et al.^2012^; Lucas et al. 2013; Růžika et al. 2015). Xylem vessels consist of a number of stacked tracheary elements (TEs) that are dead hollow cells with patterned lignified cellulose secondary cell walls (SCWs). The TEs originate through differentiation of root and shoot vascular meristem (Fukuda 2004; Milhinhos and Miguel 2013; Miyashima et al. 2013 and references therein; Devillard and Walter 2014). The differentiation passes through several stages, in the final of which the TEs undergo cell death and *post*-*mortem* autolysis (an enzymatic self-digestion of cellular content) resulting in formation of completed vessel elements (Fukuda and Komamine 1980; Fukuda 1997, 2004; Groover et al. 1997; Kuriyama 1999; Obara et al. 2001; Nieminen et al. 2004; Kubo et al. 2005; Turner et al. 2007; Jung et al. 2008; Bollhöner et al. 2013; Pesquet et al. 2013; Schuetz et al. 2013; Escamez and Tuominen 2014). During the formation of continuous vessel strands, at the place of fusion of the TEs, the primary wall at the longitudinal end of the differentiating cells adjacent to a mature TE is perforated which allows the water flow through the completed vessel conduits (Nakashima et al. 2000; Fukuda 2004). In difference to the cells in phloem vascular system, the TEs become operative after their death with a function supported by the neighbouring living cells (McCann et al. 2001; Fukuda 2004; Turner et al. 2007; Farquharson 2014).

Xylogenesis is a developmentally regulated process involving programmed cell death (PCD) (Groover et al. 1997; Fukuda 1997; Pennell and Lamb 1997; Groover and Jones 1999; Kuriyama and Fukuda 2002; Turner et al. 2007; Demura 2014; Escamez and Tuominen 2014). The PCD is a genetically determined controlled self-destruction process that is an indispensable part of the normal development and an important mechanism of survival in response to stressful environmental cues of abiotic and biotic origin. The studies on xylem differentiation and PCD occurrence in xylogenesis have been greatly potentiated since Fukuda and Komamine (1980) have introduced the xylogenic zinnia (*Zinnia elegans*) cell culture. This culture is derived from zinnia mesophyll cells that by addition of cytokinin (CK) and auxin are induced to transdifferentiate into TEs. The processes of transdifferentiation and the cellular demise are closely connected and proceed through well-concerted interplay of plant hormones, metabolic pathways, molecular and genetic factors. The recognition of the regulatory network of the TE differentiation cascade in the zinnia cell system has led to accumulation of significant amount of theoretical and experimental knowledge providing a platform for investigation of TE development in other cell cultures and xylem formation *in planta* (Basile et al. 1973; Kuriyama and Fukuda 2000; Groover et al. 1997; Roberts and McCann 2000; Dengler 2001; Kubo et al. 2005; Oda et al. 2005; Turner et al. 2007; Jung et al. 2008; Pesquet et al. 2010; Bollhöner et al. 2012; Demura 2014; Escamez and Tuominen 2014; Kondo et al. 2015; Fukuda 2016). The implementation of the basic findings into practical aspects is expected to result in creation of plants with improved xylem properties related to plant survival under conditions of water stress and for production of biofuel and biomaterials.

Xylogenic zinnia cultures contributed to most of the early findings on the hormonal and biochemical signalling, metabolic pathways and molecular and gene determinants underlying the regulation of xylogenesis. Later, similar xylogenic cultures were derived from other plant species and also *in planta* and ex vivo systems were developed. In this review we focus of the discoveries in zinnia xylogenic cell cultures but also discuss later findings in e.g. xylogenic suspension cultures of *Arabidopsis thaliana* root cells, in vivo systems of zinnia and *A. thaliana* and other models. Suggestions for further research and practical implementation of theoretical knowledge are outlined.

### PCD manifestation in xylogenesis

The classification of plant cell death is still a subject of lively debates (Supplemental File S1). According to van Doorn et al. (2011) the cell death, which is accompanied by autophagic activity such as formation of lysosome-like lytic organelles, vacuolar growth, activation of vacuolar processing enzyme (VPE), tonoplast rupture and vacuole-mediated digestion of the cellular content leaving a virtually empty cell corpse behind has been defined ‘vacuolar’ cell death. It is observed in many developmental cell death events. Cell death showing swelling of mitochondria, early rupture of plasma membrane and protoplast shrinkage resulting in a largely unprocessed cell corpse has been termed ‘necrosis’. This type of cell death may be accompanied by changes in mitochondrial membrane permeability, respiratory decline, ATP depletion and oxidative stress-related events such as enhanced production of reactive oxygen species (ROS) and reactive nitrogen species. A characteristic PCD-associated DNA laddering pattern due to enzymatic cleavage into oligonucleosomal fragments of 180 bp and multiples thereof and activation of various cell death-related plant caspase-like proteases (CLPs) that are functional homologues of caspases (the main executioners of animal PCD) may occur in both plant PCD categories. Forms of PCD expressing mixed or atypical phenotype of vacuolar and necrotic cell deaths have been classified as ‘mixed type’ or ‘modalities’ of cell death (van Doorn et al. 2011).

Developmental PCD is involved in processes related to reproduction, growth and adaptation, e.g. incompatibility during pollination of angiosperm plants, pollen tube growth, embryogenesis, aerenchyma formation in root cortex at conditions of flooding, organ shaping (e.g. formation of leaf perforations and lobes), death of root cap cells, death of cork cells that form the bark and others. The differentiation of xylem tissue is an example of developmental PCD of the vacuolar type (Greenberg 1996; Pennell and Lamb 1997; Wang et al. 1999; Fukuda 2000; Roberts and McCann 2000; Geitmann et al. 2004; Lam 2004; Bozhkov et al. 2005; van Doorn and Woltering 2005, 2010; Rogers 2006; Gunawardena 2008; Reape and McCabe 2008, 2013; Williams and Dickman 2008; Van Doorn et al. 2011; Wertman et al. 2012; Escamez and Tuominen 2014; Van Hautegem et al. 2015; van Durme and Nowack 2016).

The studies in the model system of zinnia cell culture and *in planta* have confirmed that the TE cell death expresses features mainly of vacuolar PCD (Kuriyama 1999; Obara et al. 2001; Fukuda 2004; Weir et al. 2005; Bollhöner et al. 2012, 2013; Demura 2014; Escamez and Tuominen 2014). However, in addition to vacuole expansion and collapse and cellular autolysis, typical for vacuolar cell death, also other PCD features have been observed: nucleus condensation, oxidative stress-related processes, laddering type of DNA fragmentation and activation of PCD-associated enzymes such as CLPs (Bonneau et al. 2008; Twumasi et al. 2010a; Woltering 2010; Han et al. 2012; Petzold et al. 2012 and references therein). This suggests that zinnia TE differentiation may involve signalling pathways of both vacuolar and necrotic PCD classes.

### Xylogenic zinnia cell system as a tool to study xylogenesis

Several advantages have determined the xylogenic zinnia cell culture as an efficient system for studies on xylogenesis. In this experimental model the developmental program of xylem differentiation *in planta* is well preserved in vitro which allows reliable determination of the sequence of differentiation and cell death events, observations on the morphology of the cellular organelles, identification of signalling molecules, hormonal, molecular and gene regulatory components, examination of the architecture and chemical composition of SCWs, and investigations on intercellular communication (Hosokawa et al. 2001; Pesquet et al. 2003; Tokunaga et al. 2005; Fukuda 2000; Novo-Uzal et al. 2013). The culture is initiated from leaf mesophyll cells which can be easily separated from the other leaf tissues; it comprises a homogenous cell type and expresses high potential for synchronous transdifferentiation of the mesophyll cells yielding sufficient amounts of completed TEs. The thickening patterns of SCWs (annular, spiral, reticulate and pitted) of in vitro formed TEs share the features of those *in planta*; the TEs differentiate as single cells or form small clusters of vessel-like structures resembling the xylem vessels in zinnia plant. This facilitates the observations, in difference to the complex xylem tissue (Fukuda 1996, 2004, 2016; Groover et al. 1997; Barceló 1998a, b; Pesquet et al. 2003; Gabaldón et al. 2005; Karlsson et al. 2005; Gómez Ros et al. 2006; Turner et al. 2007; Twumasi et al. 2009; Lacayo et al. 2010). In the xylogenic zinnia cell culture the phenotype of maturating TEs can be precisely determined by using various microscopy and imaging techniques that are more difficult to achieve *in planta*. The cell system is also well accessible for applications of exogenous agents to study the signalling processes during the stages of xylogenesis (Demura and Fukuda 1994; Watanabe and Fukuda 1995; Fukuda 1994; 1996; 1997, 2004; Milioni et al. 2002; Pesquet et al. 2003, 2004; Lacayo et al. 2010; Jung et al. 2008; Escamez and Tuominen 2014).

The protocol for establishment of xylogenic zinnia cell culture, introduced by Fukuda and Komamine (1980) has been applied as originally described or with modifications aiming at improving the TE differentiation rate (e.g. Church and Galston 1989; Roberts et al. 1992; Church 1993; Fukuda 1996; Ye and Varner 1996; Groover and Jones 1999; Twumasi et al. 2009, 2010a; Pesquet and Tuominen 2011; Kákošová et al. 2013; Demura, 2014). Prerequisites for realization of the xylogenic potential of zinnia cells to yield sufficient amount of differentiated TEs are the age of leaves from which the mesophyll cells are isolated, cell density, viability and health status of the culture, pH, cellular (CO) and extracellular osmolarity (EO), and medium composition, particularly the requirement for the presence of both hormones auxin and CK (Fukuda and Komamine 1980; Turner et al. 2007; Takeuchi et al. 2013). These factors can impair the transdifferentiation if not properly considered (Supplemental File S2). The basic principles of the procedure for establishment of zinnia cell system have been developed for establishing xylogenic cultures of other species such *Arabidopsis* and for elaboration of new models for induction of xylogenesis on cultured leaf segments (Kubo et al. 2005; Oda et al. 2005; Turner et al. 2007; Avci et al. 2008; Jung et al. 2008; de Rybel et al. 2009, 2016; Ohashi-Ito et al. 2010; Pesquet et al. 2010, 2013; Bollhöner et al. 2013; Schuetz et al. 2013; Escamez and Tuominen 2014; Devillard and Walter 2014; Kondo et al. 2015; Fukuda 2016).

### Stages of tracheary elements formation in zinnia cell culture

**Fig. 1:**
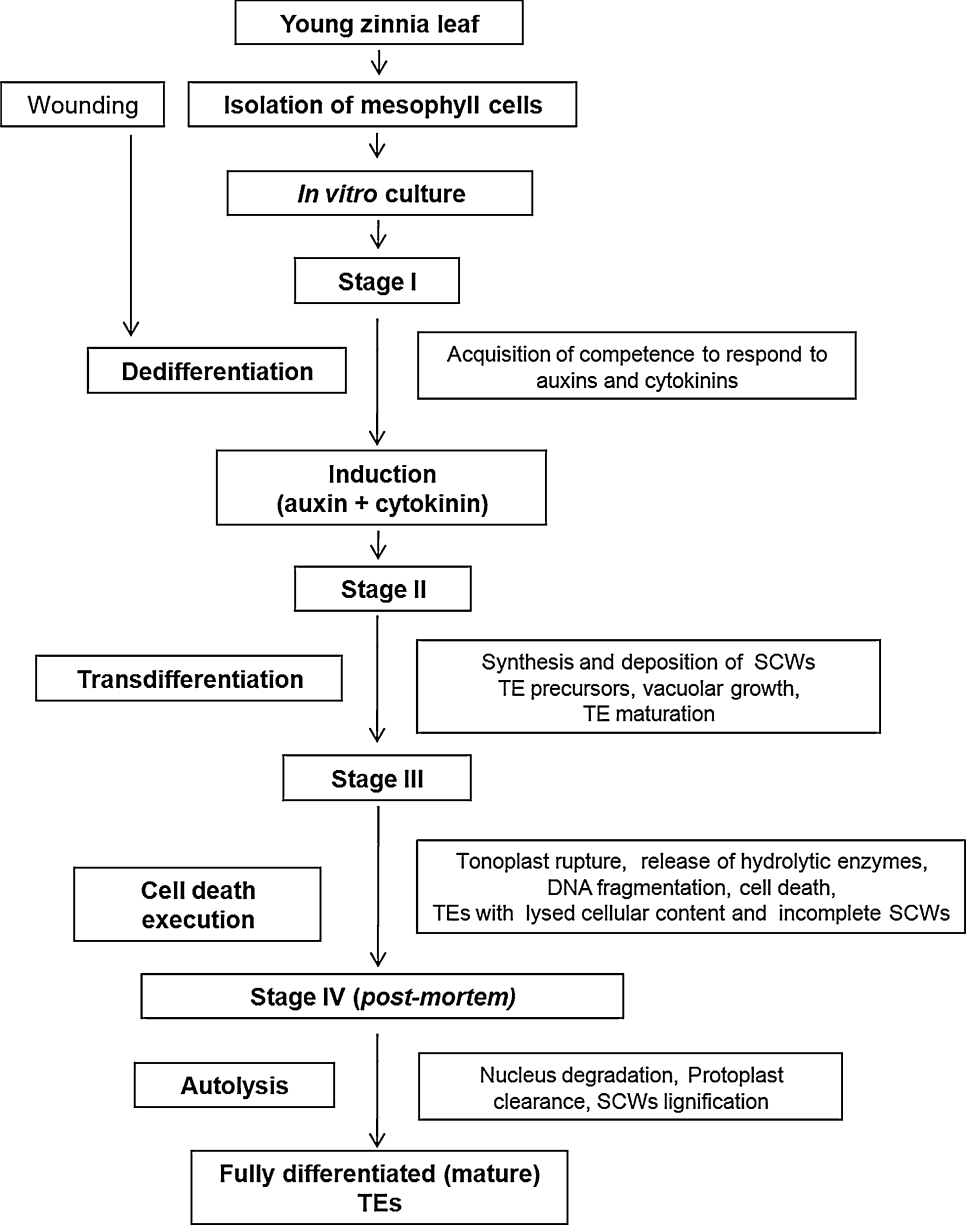
Stages of xylogenesis in zinnia cell culture. TE differentiation in zinnia in vitro proceeds through four stages: stage I: dedifferentiation of mesophyll cells and acquisition of competence to respond to auxin and cytokinin; stage II: transdifferentiation, including development of TE precursors, TE maturation and deposition of SCWs; stage III: cell death execution, continuation of SCW formation; stage IV: *post mortem* autolysis and lignification resulting in formation of completed TEs. For more detailed explanation, please refer to the text

The differentiation of xylem tissue is a paradigm of a developmental program in which differentiation, SCWs formation and cell death are tightly coupled. *In planta,* the process proceeds through a sequence of events, involving differentiation of cambial and procambial cells into TEs. This includes synthesis and deposition of SCWs material and lignification and is completed through developmentally established commitment to cellular suicide, followed by autolysis, finally resulting in generation of mature dead vessel elements capable to performing their function of water transporting system. During transdifferentiation of in vitro cultured zinnia mesophyll cells into TEs three major partially overlapping consecutive stages (Fig. 1), each associated with specific physiological state of the cells, typical morphological features, signalling interactions, molecular factors and expression of certain sets of genes have been recognized (Fukuda 1997, 2000, 2004). Stage I includes dedifferentiation of mesophyll cells which is stimulated by the wounding at isolation of the culture and during which the cells lose their ability to photosynthesize and, acquire competence for responding to auxin and cytokinin; in this stage cell division may or may not take place. Stage II is characterized by transdifferentiation, induced by exogenous supply of auxin and CK and proceeds through development of procambial initials-like cells, procambial-like cells, synthesis and deposition of SCW material, formation of immature xylem-like cells and TE precursors. Stage III is the late process of TE maturation including continuation of SCW formation and cell death execution, the latter accompanied by vacuole expansion, disruption of tonoplast integrity followed by release of endonucleases, proteases and other hydrolytic enzymes from the vacuole, partial lysis of the cellular content and DNA fragmentation.

It has been assumed that final TE cell death execution and autolysis following the vacuole burst resulting in complete digestion of the protoplast and the nucleus are a common expression of the cell death process occurring in stage III (Fukuda 1996; Greenberg 1996; Fukuda et al. 1998). However, it was also suggested that the final stage of PCD may be split in two consecutive phases—cell death execution and autolysis, the latter of which is responsible for complete protoplast elimination (Mittler and Lam 1995; Jones and Dangl 1996; Groover et al. 1997; Groover and Jones 1999; Nakashima et al. 2000; Jones 2001; Kozela and Regan 2003). Escamez and Tuominen (2014) described the cell death and autolysis of TEs as two separate consecutive phases in stage III, in the first of which TE cells die but autolysis resulting in clearance of organelles remnants to form hollow dead mature TEs occurs *post mortem*, within few hours after cell death (Fig. 1). When the vacuole collapses, the cell is dead but the released lytic enzymes proceed to degrade the protoplast debris. Moreover, the *post*-*mortem* stage (we suggest to be determined as stage IV) is featured by an active process of SCWs lignification which is non-autonomous and is supported by substances delivered from neighbouring living cells both in zinnia in vitro and *in planta*, and in other cell cultures and *in planta* systems such as differentiating xylem in *Arabidopsis* roots and hypocotyls of *Phaseolus vulgaris* (Smith et al. 1994; Hosokawa et al. 2001; Fukuda 2004; Tokunaga et al. 2005; Avci et al. 2008; Bollhöner et al. 2012, 2013; Novo-Uzal et al. 2013; Pesquet et al. 2010, 2013).

**Fig. 2:**
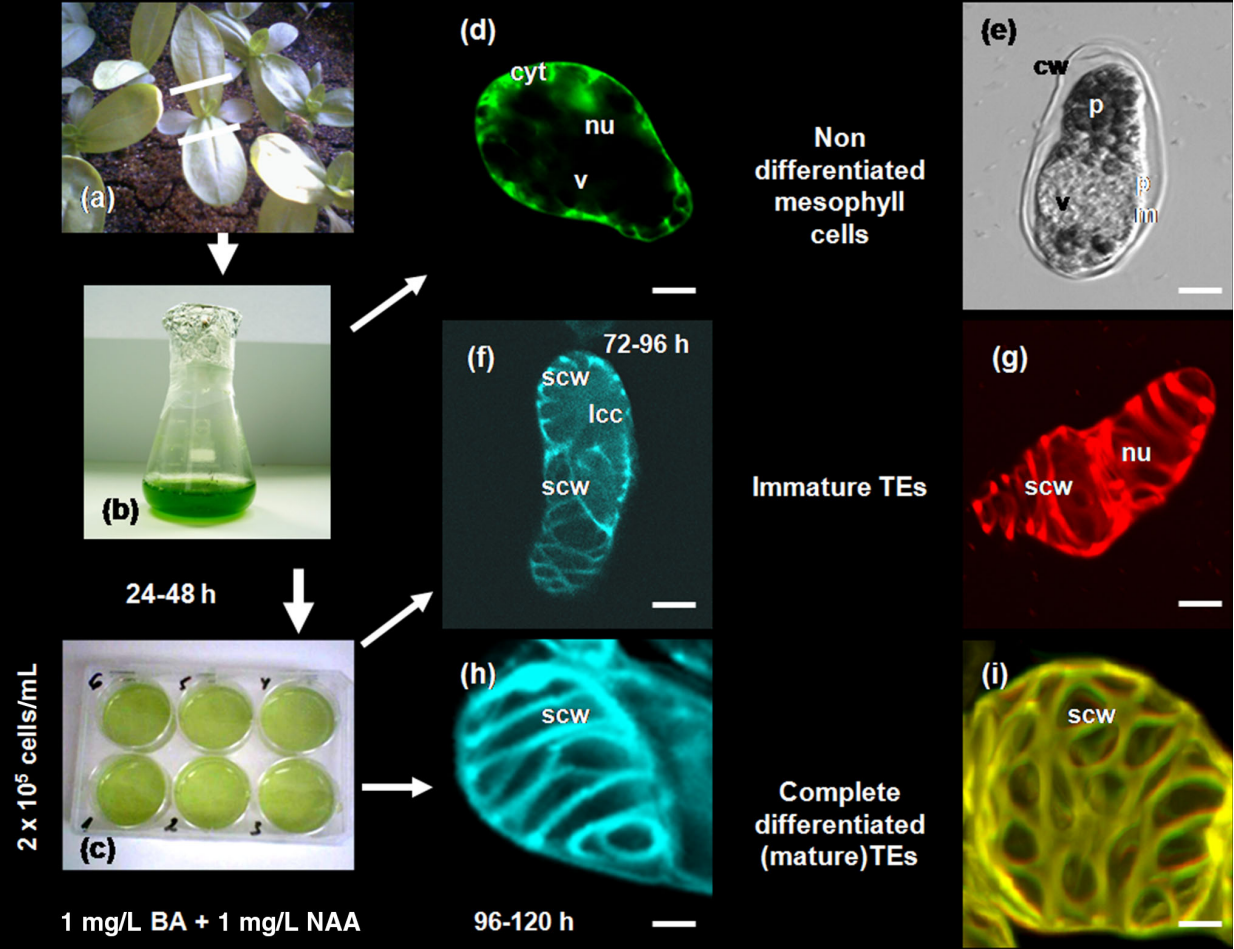
Zinnia culture isolation and induction and cellular morphology during transdifferentiation and cell death progression of TEs in xylogenic zinnia cell system. **a** Cell suspension was started from the first pair of true leaves of 14 days’ old seedlings of *Zinnia elegans*, cv. Envy, as described in Tuwmasi et al. (2010a, b). **b** Isolated cells were kept in 100 mL sterile flasks. **c** 24–48 h after isolation the culture was diluted to obtain cell density 2.10^5^ cells/mL, 3 mL suspension was transferred to 6-well plates, supplemented with 1 mg/L BA and 1 mg/L NAA and further used for experiments with chemical treatments. **d** Fluorescein diacetate (FDA) stained living mesophyll cell; visible are intact FDA positive cytoplasm, diffuse nucleus and FDA negative intact vacuole. **e** Transmission light microscopy image of dead mesophyll cell showing features of necrotic cell death, i.e. shrunken protoplast and plasma membrane retracted from the cell wall. **f** Calcofluor White (CFW) labeled immature TEs; incomplete SCWs and amorphous cellular content are distinguishable. **g** Propidium iodide (PI) labeled immature TE; SCWs are partially completed, and cellular content is lysed but the compact PI positive nucleus is still preserved. **h** Calcofluor White stained mature TE; visible are completed secondary cell walls (SCW) of an empty hollow cell, after autolysis of the cellular content; the nucleus has disappeared. **i** Autofluorescence from SCWs of a mature TE. The micrographs were collected by using a TCS SP2 AOBS CLSM system (Leica-Microsystems GmbH, Mannheim, Germany) mounted on an inverted Leica DM IRE2 microscope. Three different laser wavelengths (405, 488 and 561 nm) were employed for excitation, three emission channels for fluorescence imaging and one separate channel for non-confocal transmission imaging. Overlays and orthogonal projections were made using the Leica Confocal software. Cell wall (cw), lysed cellular content (lcc), nucleus (nu), protoplast (p), plasma membrane (pm), secondary cell wall (scw), vacuole (v).* Scale bars* 20 µm

Various studies have demonstrated that the zinnia cell system is well appropriate for assessment of the morphological appearance and cell death progression in the consecutive stages of TE development by means of high resolution microscopy such as light, fluorescent and confocal laser scanning microscopy (CLSM), transmission electron microscopy (TEM), atomic force microscopy (AFM), synchrotron radiation-based (SR)-FTIR spectromicroscopy and other techniques (Supplemental File S3). Our own experience supported the suitability of some of the labelling methods for identification of cell death features in in vitro differentiating zinnia TEs (Fig. 2). The labelling techniques used for the studies with zinnia are applicable also for similar purposes in other xylogenic systems.

The use of chemical agents interacting with various pathways (known as pharmacological analysis) is well established approach for investigating the cellular signalling in vitro and in *planta*. This experimental tool has been widely applied to study the transdifferentiation**/**PCD signalling in xylogenic zinnia cell culture (Watanabe and Fukuda 1995) and has helped to reveal important factors involved in the control of xylogenesis also in other in vitro models (Supplemental File S4).

### Regulation of xylogenesis in zinnia cells

Xylogenesis in zinnia cell system proceeds through a well-coordinated program in which a number of regulatory pathways are integrated. A network of signalling interactions, metabolic pathways and gene and transcriptional factors involved in zinnia differentiation and PCD in vitro has been described also during xylem genesis in other cell and *in planta* model systems such as *Arabidopsis thaliana*, *Populus, Pinus, Phyllostachys bamboo, Musa banana* and others (Fukuda and Komamine 1980; Iwasaki et al. 1986; Aloni 1987; Church and Galston 1989; Church 1993; Fukuda 1992, 1994, 1996, 1997, 2000, 2004, 2016; Kalev and Aloni 1998a, b; Yamamoto et al. 1997, 2001, 2007; Krishnamurthy et al. 1999; Kuriyama and Fukuda 2000; McCann 1997; Sachs 2000; Demura et al. 2002; Pesquet et al. 2003, 2004; 2013; Nieminen et al. 2004; Kubo et al. 2005; Turner et al. 2007; Jung et al. 2008; Motose et al. 2009; Pesquet and Tuominen 2011; Ogita et al. 2012; Bollhöner et al. 2012; Milhinhos and Miguel 2013; Escamez and Tuominen 2014; Aloni 2015; Demura 2014; Didi et al. 2015; Negi et al. 2015; Růžika et al. 2015; Kondo et al. 2015).

### Wounding-associated hormonal regulation

In plant tissues the wounding induces a cascade of signalling events culminating in various defence responses and PCD. The nature of wound signals generated at the primary site of physical injury and transmitted toward neighbouring cells and/or at longer distance has not yet been fully identified. Among the candidates for this role are ROS, jasmonic (JA) and salicylic (SA) acids, ethylene and electrical waves (León et al. 2001, and references therein). Ryan (2000) suggested that the wounded leaf cells may excrete the peptide systemin which binds to a transmembrane receptor of neighboring cells and initiates a sequence of signal transduction events involving Ca^2+^ influx, MAP kinases, phospholipase A2, linoleic acid and octadecanoid pathway resulting in the synthesis of JA. The latter in turn may amplify the wound signal through enhancing prosystemin gene expression and the expression of other genes contributing to differentiation of xylem cells to build new xylem routes for bypassing the injured leaf area.

Transdifferentiation of in vitro cultured zinnia mesophyll cells is stimulated by wounding during the isolation of the cells (Kuriyama and Fukuda 2000; Fukuda 1997, 2000). Matsubayashi et al. (1999) found that in zinnia culture with low cell density, which suppresses the transdifferentiation, the addition of the sulfated peptide hormone phytosulfokine-*α* (PSK) or cultivation of mesophyll cells in conditioned medium recovered the process of TE formation. This indicated that wounded cells may produce and release PSK into the medium thus promoting the transdifferentiation. Through the use of specific inhibitors and gene expression analysis, a role of PSK in the wound response has been confirmed. This hormone accumulates in the early stages after culture initiation and subsequently in the last stage of TE development. Inhibition of PSK action with chlorate (KClO_3_), an inhibitor of *Tyr*-*O*-sulfation of a PSK precursor, significantly suppressed the process of transdifferentiation (Motose et al. 2001a, b, 2009). It was established that in response to wounding PSK precursor gene *ZePSK1* transcripts transiently accumulate in 24 h cultures and again at the entry into the final differentiation stage, whereby *ZePSK1* expression was dependent on brassinosteroids (BRs) (Yamamoto et al. 1997, 2001; Iwasaki and Shibaoka 1991; Motose et al. 2009). Such interaction was supported by findings showing that uniconazole and brassinazole, which inhibit the synthesis of BRs can repress transdifferentiation in the early and late stages (Iwasaki and Shibaoka 1991; Yamamoto et al. 1997, 2001).

Phytosulfokine performs multiple regulatory functions in plants and is suggested to integrate the growth and defence signals (Sauter 2015). Microarray analysis revealed that in the xylogenic zinnia cell culture, in the presence of PSK, a number of stress-induced genes encoding for e.g. chitinases, phenylalanine ammonia-lyase (PAL), 1-aminocyclopropane-1-carboxylic acid synthase (ACS), receptor-like protein kinases and proteinase inhibitors are down-regulated. This suggests that PSK-associated signalling might be involved in the suppression of stress response. Taken together with elevated level of *ZePSK1* transcripts after wounding (Yamamoto et al. 1997, 2001; Iwasaki and Shibaoka 1991, Yoshida et al. 2009) these findings indicated that PSK possibly participates in mitigation of the wound effect through stimulation of transdifferentiation thus leading to xylem tissue regeneration (Motose et al. 2009). It remains to be elucidated whether PSK directly stimulates gene expression responsible for TE differentiation or acts through enhancing metabolic, transcriptional and translational activities that are commonly responsible for TE differentiation and for increasing the cell density in the culture by promoting the cell division (Matsubayashi et al. 1999).

The wound response has been found associated with the expression of wound-inducible genes encoding proteinase inhibitors and ethylene (O’Donnell et al. 1996; Ryan 2000). In isolated zinnia mesophyll cells during dedifferentiation (stage I) these genes are upregulated and during transdifferentiation (stage II) are downregulated (Fukuda 1996, 1997). Such findings suggest that the wound signal may play a dual role: to potentiate a defence reaction by preventing the proteolysis in mesophyll cells and further, by self-amplification to promote gene expression or post-translational activation of proteases involved in the later process of TE cell death (Kuriyama and Fukuda 2000).

In xylem tissue JA or methyl jasmonate (MeJa) may amplify wound signals by inducing the expression of genes involved in development of the vessel elements (Kuriyama and Fukuda 2000). *In planta* JA has been suggested to trigger cambium cell division, which in turn might be related to an effect on xylem formation (Sehr et al. 2010). By expression profiling of hormone-related gene homologues in xylogenic zinnia cell culture Yoshida et al. (2009) established that the genes *Z8696*, *Z8562* and *Z7649* that are associated with JA synthesis and the genes *Z8771* and *Z7791* (involved in JA signalling) are expressed in stage I of the process.

*Cis*-abscisic acid (ABA) is suggested to play signalling role in association with the wound response in xylem (Kuriyama and Fukuda 2000). An expression of ABA-inducible homeobox gene has been detected in *Arabidopsis* vascular bundles (Vicient et al. 2000) and expression of ABA-regulated gene encoding for proteins in embryo procambial tissue of carrot has been reported (Wurtele et al. 1993). The contribution of both JA and ABA to wound signalling has led to suggestion that they might be involved in the transduction of wound signals during zinnia xylogenesis in vitro (Fukuda and Komamine 1985; Kuriyama and Fukuda 2000). And indeed, in zinnia culture Yoshida et al. (2009) detected ABA biosynthesis-related genes *Z4493* and *Z6166* and ABA-responsive element binding protein Z5783 that are homologues to *Arabidopsis* (Uno et al. 2000). In similarity to JA, these genes have been expressed in stage I of xylogenesis. The same authors suggested an interaction of auxin with JA and ABA. They found that JA- and ABA-related genes were downregulated when the auxin NAA was added to the zinnia culture. Gene expression analysis revealed that the JA- and ABA-related genes in zinnia are homologues to *Arabidopsis* genes involved in JA and ABA biosynthesis and signalling. It was suggested that JA and ABA might indirectly contribute to xylem differentiation and that the induction of the culture with auxin, which stimulates transdifferentiation, might interrupt the progression of stage I.

Although not yet well known, it is suggested that during stage I, ethylene, SA, JA and PSK might operate in conjunction. Motose et al. (2009) described a potential communication of PSK signalling with other pathways. In PSK treated zinnia cell culture in the absence of auxin and CK, they found that several stress-responsive genes such as those encoding enzymes in phenylpropanoid pathway, chitinases, receptor-like protein kinases, ACS and other defense-associated proteins were downregulated. The suppression of genes from ethylene biosynthesis and PAL pathway suggested that this may result in suppression of SA and ethylene production, thus affecting the mediation of wound-induced signalling in which SA and ethylene are supposed to play a role. The results point to a role of PSK in mitigation of the wound response in the early stages of TE differentiation. This assumption was supported by additional experiments involving the application of stress-inducible hormones in conjunction with PSK. Jasmonic acid and MeJa suppressed the PSK-induced TE formation, whereas in the presence of SA, acetyl salicylic acid, ethylene precursor 1-aminocyclopropane-1-carboxylic acid (ACC) and ethylene releasing compound 2-chloroethylphosphonic acid (ethephon), the percentage of formed TEs was almost unaffected (Motose et al. 2009).

### Auxin and cytokinin

Cytokinin and auxin are compulsory required for induction of transdifferentiation of zinnia mesophyll cells. *In planta* the polar auxin flow ensures the continuous formation of vascular tissue (Sachs 2000). The acropetal auxin transport drives the hormone from apical meristem, where it is synthesized, toward procambial cells resulting in their differentiation to form mature vessel strands. In the case of wounding, auxin transport is interrupted leading to disturbed mode of xylem development (Kuriyama and Fukuda 2000; Fukuda 2004; Mattsson et al. 1999 and references therein).

The molecular components of auxin perception in transdifferentiating zinnia cells are still poorly understood. Some of the transcription factors and expressed genes involved in auxin-flow-dependent procambial cell differentiation are described for *Arabidopsis* (Fukuda 2004; Milhinhos and Miguel 2013; Demura 2014; Fàbregas et al. 2015). Studies suggested that auxin signalling during zinnia TE differentiation in vitro might interfere with galactoglucomannan oligosaccharides (GGMOs), which in a concentration dependent manner may act as potential competitive antagonists of auxin (Kákošová et al. 2013). Low auxin but not low CK concentrations in a xylogenic medium supplemented with GGMOs did not disturb the normal pace of transdifferentiation process but the portion of MX-like TEs was higher than that of PX-like TEs, in contrast to the induced control culture lacking GGMOs. The number of R-like TEs was not affected. The authors assumed that GGMOs could be involved in MX-like TE formation through auxin signalling pathway. Auxin was suggested to repress the wound response thereby promoting the early stages of TE differentiation. This was supported by microarray analysis of in vitro transdifferentiating zinnia cells, reported by Yoshida et al. (2009). The authors identified cDNAs corresponding to proteins involved in auxin biosynthesis, metabolism, transport, and cDNAs acting as transcription factors homologues to *Arabidopsis*. Early auxin response genes were identified 0.7 h after addition of NAA. The genes expressed 4 h after NAA treatment were homologous to *VASCULAR*-*RELATED NAC*-*DOMAIN PROTEINs (VNDs)* which encode NAC-domain transcription factors, found in procambial cells (Kubo et al. 2005). Other genes corresponded to *HD*-*ZipIII* homeobox genes that accumulate in procambial xylem precursor cells and in developing TEs (Ohashi-Ito et al. 2005). Additionally, in the same set of experiments genes homologues to auxin transporter proteins, the influx carrier AUX1 and the efflux carrier proteins of PIN family were upregulated 4 h after addition of NAA. These data substantiated the role of auxin in the early stages of TE development (Yoshida et al. 2009). However, the expression of ethylene-related genes was almost unaffected when the zinnia cell suspension was supplemented with auxin (Yoshida et al. 2005, 2009) which indicated that in the early transdifferentiation stage auxin might exert its effects independently on ethylene.

Cytokinins are responsible for vascular development through promoting cambium and procambium cell proliferation and acting in crosstalk with auxin (Fukuda and Komamine 1980; Church and Galston 1989; Aloni 1993; Church 1993; Fukuda 1997, 2000, 2004; Kuriyama and Fukuda 2000; Pesquet et al. 2013; Milhinhos and Miguel 2013). Bishopp et al. (2011) showed that in *Arabidopsis* root vasculature the cells designated to become protoxylem exhibit high auxin and low CK levels, whereas the procambial cells exhibit high CK and low auxin levels. Fukuda (2004), Mähönen et al. (2006), Bishopp et al. (2011) and Milhinhos and Miguel (2013) reported arguments suggesting that in procambial cells the coordinated signalling by CK and auxin induces the expression of genes that encode for components responsible for the maintenance of procambial activities. The auxin-signalling pathway might involve gene expression of auxin-response factors, such as MONOPTEROS (MP), that also function as transcriptional activators and the gene expression of their repressors, the AUX/IAA proteins. It has been suggested that CK might be perceived by the CYTOKININ RESPONSE1/WOODEN LEG/ARABIDOPSIS HISTIDINE KINASE4 (WOL/CRE1/AHK4) CK receptor and ARABIDOPSIS HISTIDINE KINASE2 (AHK2) and AHK3 that activate a phosphorylation cascade in which histidine phosphotransfer proteins (AHPs) activate type-B ARABIDOPSIS RESPONSE REGULATORS (type-B ARRs) in the nucleus. In turn, these factors might function as transcriptional activators of procambium genes including the genes of their repressors, the type-A ARRs, finally resulting in CK responses. The activators and repressors in auxin- and CK-signalling pathways might control the temporal CK/auxin effects (Kieber and Schaller 2010; Bishopp et al. 2011). In the zinnia cell system, cytokinin is suggested to promote dedifferentiation of mesophyll cells prior to transdifferentiation into TEs (Turner et al. 2007). The interplay of auxin and CK in the early stages of transdifferentiation of zinnia cultured cells was supported by the finding that 4 h after the administration of NAA the expression of cytokinin oxidase homologue was enhanced which indicated that auxin might act through activating this enzyme and in this way reducing the CK level (Yoshida et al. 2009).

### Brassinoid-associated regulation

It was suggested that BRs contribute to the early transdifferentiation processes in zinnia in vitro (Yamamoto et al. 1997; Motose et al. 2001a, b; Fukuda 2004). This was confirmed by results showing that in xylogenic zinnia cell culture, during stage II, transcripts of genes involved in BR synthesis accumulate in procambium-like cells that differentiate into xylem precursor cells (Yamamoto et al. 2007). The presence of auxin and CK in inductive zinnia medium is considered sufficient to evoke *de novo* synthesis of the endogenous BR castersteron which is secreted out of the cells and may function as intercellular signal in the early stage of transdifferentiation and in the terminal stage of cell death (Yamamoto et al. 2001; Motose et al. 2001a, b, 2009). Yoshida et al. (2009) showed that in stage II auxin might affect BR metabolism in a sophisticated manner. They demonstrated that NAA promoted the synthesis of brassinolide intermediates, but suppressed its biosynthesis and stimulated enzymes that inactivate this BR. The authors suggested that the low levels of active BRs may be a mechanism for suppressing the immediate transdifferentiation of mesophyll cells into TEs.

The synthesis of BRs might be stimulated by the hormone PSK produced in response to wounding (Yamamoto et al. 1997, 2001; Iwasaki and Shibaoka 1991; Motose et al. 2009). The involvement of BRs in the control of transdifferentiation has been demonstrated by a TE differentiation-specific increase of transcripts for *ZeDWF4* (*DWARF 4*) and zinnia caroxypeptidase (*ZeCPD1*) genes. In *Arabidopsis*, these genes are suggested to encode for enzymes in BRs synthesis pathway (Mathur et al. 1998). In xylogenic zinnia culture the transcripts drastically accumulated in stage II when PC-like cells are produced which points out that BRs might be synthesized in PC-like TEs and might initiate the progression to stage III (Fukuda 2004; Yamamoto et al. 2007).

Interaction of auxin, CK and BRs has been suggested to influemce the activity of basic peroxidase izoenzyme ZePrx that is involved in lignin biosynthesis in differentiating xylem in zinnia seedlings. Treatment of the seedlings with auxin and CK induced ZePrx and metaxylem differentiation during seedling secondary growth whereas the exogenous application of BRs exerted an opposite effect. The results indicated that these three hormones might also control ZePrx participation in xylem lignification (Gutiérres et al. 2009).

### Gibberellin-related signalling

Gibberellin (GA3) is another plant growth regulator (PGR) implicated in TE differentiation in xylogenic zinnia cultures. Gibberellin effects are generally linked to cell elongation where it cooperates with auxin. It is thought that endogenous GA3 contributes to lignification (Tokunaga et al. 2006). In conditioned control medium Tokunaga et al. (2006) detected high levels of lignin precursors that were strongly reduced in medium from GA3 treated cells. They suggested that GA3 may act through activating the polymerization of lignin precursors. Addition of GA3 before hormonal induction of the culture caused a delay of TE differentiation suggesting that GA3 might exert an inhibitory effect during the early stage of transdifferentiation. A lignification-associated interaction of auxin with GA3 signalling has been assumed based on the findings that in zinnia cell cultures supplemented with NAA, GA3 synthesis genes were upregulated (Yoshida et al. 2009). Tokunaga et al. (2006) hypothesized that the effect of GA3 on the retardation of transdifferentiation in the early stages of zinnia xylogenesis in vitro might be attributed to a GA3-mediated delay of wound response. *In planta*, GA3 might, in cooperation with auxin and ethylene, be able to modulate the establishment of TE cell polarity in order to ensuring proper TE morphogenesis in vascular tissue (Aloni 1987; Kalev and Aloni 1998a, b).

### Nitric oxide, PAs and ethylene dependent signalling

Nitric oxide (NO) is a bioactive signalling factor contributing to processes related to SCW lignification and transdifferentiation of zinnia vessel elements (Gabaldón et al. 2005; Gómez Ros et al. 2006). This ubiquitous gaseous molecule is involved in the mediation of diverse physiological processes, abiotic and biotic stress responses and PCD. It can interact with cysteine-thiol groups and inactivate proteins through S-nitrosylation or through inactivating enzyme co-factors such as ferrous ion. In cooperation with ROS, NO and reactive nitrogen species may exert antioxidant and pro-oxidant as well as cell death-protecting or death-promoting effects (Delledonne et al. 1998; Neill et al. 2003; Wendehenne et al. 2004; Iakimova and Woltering 2015). The contribution of NO to lignification and cell death of zinnia TEs has been documented by microscopy observations with NO-sensitive fluorescent probes and pharmacological studies with NO releasing and scavenging agents (Supplemental File S3, Supplemental File S4) (Gabaldón et al. 2005; Gómez Ros et al. 2006; Novo-Uzal et al. 2013). Recently, in differentiating xylem of *Populus* roots, *in planta*, Bagniewska-Zadworna et al. (2014) established contribution of NO signalling to the onset of cell differentiation and further at all stages of TEs maturation but not in the mature vessel elements. As possible targets of NO action, transcription factors and/or activity of some of the enzymes in lignin biosynthesis have been suggested (Gabaldón et al. 2005; Gómez Ros et al. 2006). However, to verify the NO effects more profound molecular analysis and gene transcriptional profiling are necessary.

Polyamines (PAs) are thought to exert effects on cell division, vascular cambial activity, cell differentiation and cell death (Muñiz et al. 2008 and references therein). During zinnia TE development in vitro the PAs are suggested to prevent and/or delay the premature cell death of the TEs in the early transdifferentiation stages, thus allowing the growth of TEs with larger dimensions (Muñiz et al. 2008; Milhinhos and Miguel 2013). The effect of PAs on cell death has been attributed to their ability to protect membrane stability by blocking the ion leakage from vacuoles and preventing the changes in mitochondrial membrane permeability. Due to their slight cationic charge PAs may also function as potent ROS scavengers thus reducing the severity of oxidative stress (Muñiz et al. 2008 and references therein). The PAs spermine and thermospermine may possibly function as limiting factors that might regulate the levels of endogenous auxin or transcription factors responsible for auxin synthesis and auxin-dependent differentiation response and by such mechanism may control the timing of differentiation. Spermine and thermospermine synthase are encoded by a putative *Arabidopsis ACAULIS 5* (*ACL5*) gene (Hanzawa et al. 2000). In *acl5* mutants the hypocotyls did not develop xylem tissue. The expression of *ACL5* in zinnia cultured cells occurred before the onset of transdifferentiation and corresponded to the activation of the same gene established in protoxylem cells in *Arabidopsis* hypocotyls. It was suggested that also *in planta* PAs might prevent premature death of developing vessel elements thus allowing complete expansion and structuring of SCW patterning (Muñiz et al. 2008).

The studies have indicated that ethylene is involved in the signalling of zinnia TE differentiation (Pesquet and Tuominen 2011; Pesquet et al. 2013). Ethylene, PAs, and NO are proposed to act as transmitters of wound-activated transdifferentiation/PCD signalling in the early stages and to operate in interplay for exerting effects in the later stages of cell death (Gabaldón et al. 2005; Muñiz et al. 2008; Pesquet and Tuominen 2011; Yoshimoto et al. 2012; Milhinhos and Miguel 2013; Pesquet et al. 2013). Ethylene and PA synthesis is intersected at the level of their common precursor s-adenosylmethionine, but to which extent the metabolic pathways of these hormones might crosstalk during zinnia TE differentiation is still not well understood. Nitric oxide production has been found associated with the early transdifferentiation process and with the stages immediately preceding the process of SCW formation and cell autolysis (Gabaldón et al. 2005). This gaseous molecule is also presumed to link PAs and ethylene signalling with cell death (Milhinhos and Miguel 2013 and references therein).

The findings on hormone interactions soundly demonstrate the complexicity of the processes of transdifferentiation and PCD in zinnia in vitro and point out that for better elucidation of these regulatory mechanisms further studies are necessary, especially concerning the molecular targets of the hormonal signals.

### Oxidative stress-related regulation

The TE development in zinnia culture occurs in highly oxidative state (Barceló 1998a, b, 1999; 2005; Gómez Ros et al. 2006; Novo-Uzal et al. 2013), the level of which is dependent on ROS production and their detoxication by the cellular enzymatic and non-enzymatic antioxidant system. The involvement of ROS and especially H_2_O_2_ in PCD is well established (Levine et al. 1994). In the differentiation of xylem tissue, H_2_O_2_ is required for lignification (Novo-Uzal et al. 2013 and references therein). It is involved in peroxidase-mediated oxidative polymerization of cinnamyl alcohols to lignins and in the reinforcement of the cell wall through participating in cross-linking of cell wall proteins (Ogawa et al. 1997; Olson and Varner 1993; Levine et al. 1994; Barceló 1998a, b and references therein; Liu et al. 1999). The observations indicated that in the cell culture and in zinnia stems the living non-differentiating cells produce ROS before and at the beginning of lignification of SCWs. The early H_2_O_2_ synthesis in the vital cells was suggested necessary for lignification in the earlier and later, including *post mortem*, stages of SCWs formation. The H_2_O_2_ released from the living cells is supplied to differentiating TEs through the intercellular spaces (Olson and Varner 1993; Ferrer and Barceló 1999; Barceló 1998a, b, 2005; Weir et al. 2005; Gómez Ros et al. 2006).

It was suggested that H_2_O_2_ in differentiating zinnia cells is generated by a dual mechanism—through membrane-localized NADPH oxidase (an enzyme responsible for conversion of O_2_^−^ to H_2_O_2_) and/or through basic peroxidase (Barceló 1998a, 1999, 2005; Novo-Uzal et al. 2013 and the references cited therein). The question whether lignification in cultured TEs and in the xylem in zinnia stems is under the same enzymatic control, especially with respect to peroxidase-mediated polymerization of ρ-hydroxycinnamyl alcohols into lignins, has been approached by experiments in both systems. A cationic peroxidase was purified from differentiating TEs (Sato et al. 1993, 1995) and confirmed by microarray analysis (Demura et al. 2002). The authors reported gene expression of basic peroxidase at the time of SCW lignification both in vitro and *in planta*. The existence of a sole basic peroxidase located in the cell wall of xylem elements in zinnia hypocotyls, stem and leaves and in in vitro differentiating TEs was substantiated by López-Serrano et al. (2004) and by Fukuda and Komamine (1982). They proposed peroxidase as a marker of TE lignification in zinnia in vitro and in lignifying xylem *in planta*. A second xylem H_2_O_2_ producing pathway was suggested in a study based on addition of peroxidase inhibitor salicylhydroxamic acid which resulted in suppressed TE development (Karlsson et al. 2005). The function of a NADPH oxidase-like enzyme in lignifying zinnia xylem cells was supported by pharmacological studies involving administration of a range of NADPH oxidase inhibitors such as pyridine, imidazole, quinacrine and diphenylene iodonium. Treatment of zinnia xylem tissue with these chemicals led to decreased H_2_O_2_ production and disturbed lignification (Barceló 1999; Gómez Ros et al. 2006). Participation of O^2−^ dependent laccases in the production of lignin monomer radicals has also been demonstrated (Ranocha et al. 1999; Boerjan et al. 2003; Barros et al. 2015).

A non-enzymatic factor implicated in the regulation of cellular redox homeostasis is the peptide glutathione (GSH). During isolation, the zinnia mesophyll cells are exposed to wound-induced oxidative stress which stimulates the dedifferentiation. In experiments of Henmi et al. (2005) in zinnia xylogenic cell cultures, elevated endogenous level of glutathione disulfide (GSSG)—an oxidized from of GSH has been detected. The authors reported that exogenous application of GSH suppressed TE differentiation whereas the addition of GSSG increased the number of differentiated TEs if applied at early stage of cell culturing. This suggested that the balance between GSH and GSSG might be involved in the regulation of the initial stages of TE development (Henmi et al. 2005).

### Calcium and other signalling molecules

Various studies, mostly pharmacological analyses, have indicated that the regulation of TE differentiation in zinnia culture is dependent also on other signals and pathways such as Ca^2+^/CaM signalling in a relatively early stage of transdifferentiation prior to the onset of SCWs deposition, phenylpropanoid pathway contributing to lignin production, heterotrimeric G-proteins, lipid-derived signals, protein phosphorylation and others (Ingold et al. 1990; Suzuki et al. 1992; Groover et al. 1997; Barceló 1999; Groover and Jones 1999) (Supplemental File S4).

### Contribution of proteases, nucleases and other proteins

**Fig. 3:**
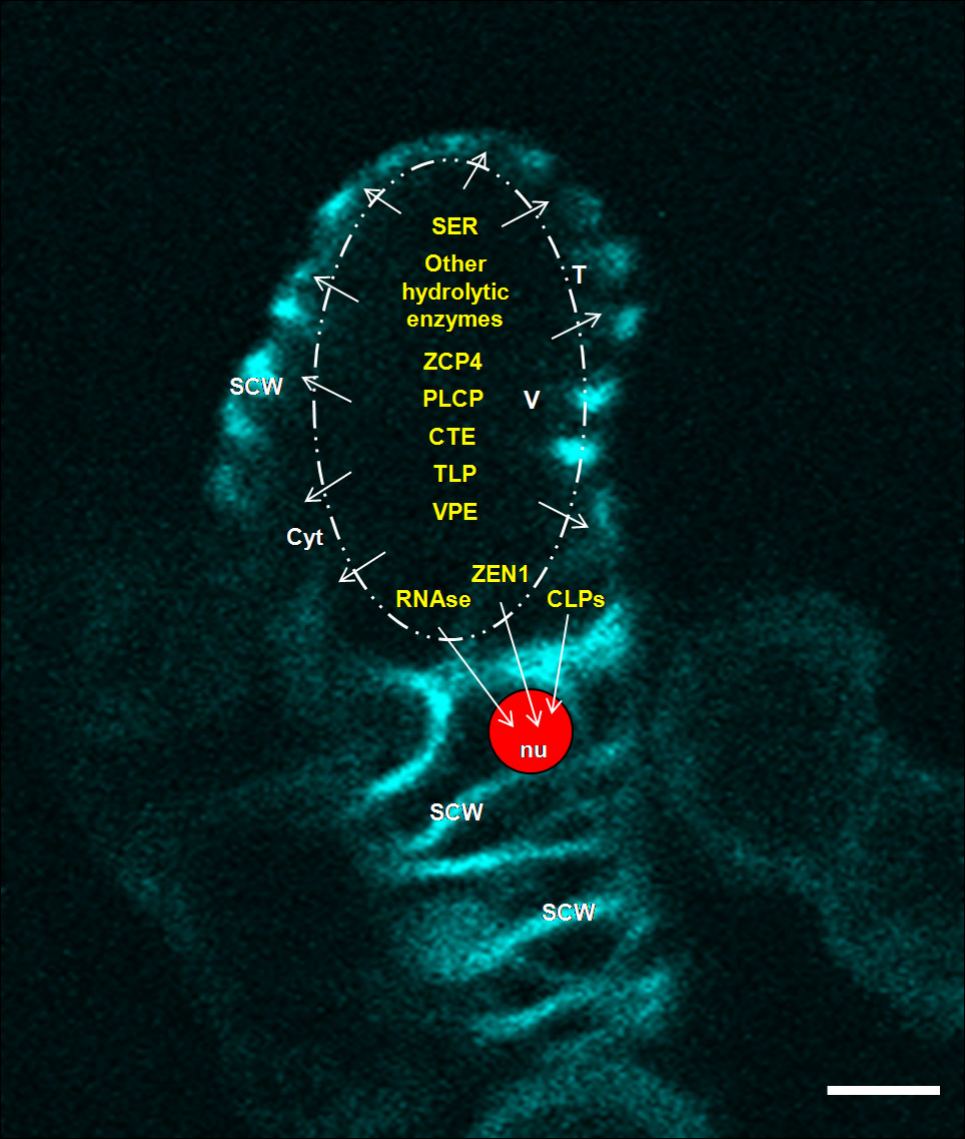
Suggested contribution of lytic enzymes to cell death execution of in vitro generated zinnia tracheary elements. In stage III of xylogenesis in in vitro cultured zinnia cells the tonoplast ruptures and various lytic enzymes are released from the vacuole resulting in autolytic digestion of the protoplast and DNA fragmentation. The SCWs are partially completed, nucleus is condensed. Caspase-like proteases from cytoplasm and oligonucleases (DNAse ZEN1 and RNAses) from the vacuole contribute to DNA cleavage in the nucleus; SER and CTE might be involved in proteolysis of cytoplasmic proteins; TLP might participate in tonoplast collapse and autolysis; PLCP participates in autolysis and SCW deposition; VPE—a plant caspase-1-like protease might contribute to tonoplast rupture and vacuole-mediated digestion of the cellular content; other hydrolytic enzymes might also be released from vacuole. Background image—a dead tracheary element in stage III of transdifferentiation; incomplete and completed SCWs are distinguished following Calcofluor White labelling. The image is taken by a TCS SP2 AOBS CLSM system mounted on an inverted Leica DM IRE2 microscope and by using the Leica Confocal software as described in the legend of Fig. 2. Caspase-like protease (CLP), cathepsin-like endopeptidase (CTE), cytoplasm (cyt), nucleus (nu), papain-like cysteine protease (PLCP), secondary cell wall (SCW), serine protease (SER), thrombin-like protease (TLP), tonoplast (T), vacuolar processing enzyme (VPE), zinnia cysteine protease 4 (ZCP4).* Scale bar* 10 µm

Enzymes such as serine and cysteine proteases and nucleases (Fig. 3) have been identified and found to operate during cell death execution and in the earlier stages of zinnia xylogenesis in vitro (Thelen and Northcote 1989; Beers and Freeman 1997; Ye and Varner 1996; Fukuda 2000; Kubo et al. 2005; Pesquet et al. 2004). Most of these activities are considered as markers of xylogenesis (Fukuda 1996, 1997, 2000, 2004; Groover et al. 1997; Kuriyama 1999; Obara et al. 2001; Milioni et al. 2002; Demura et al. 2002; Pyo et al. 2004, 2007; Pesquet et al. 2004; Endo et al. 2008; Demura 2014).

Zinnia endonuclease 1 (ZEN1) which is S1-type nuclease was shown to play a central role in nuclear DNA degradation in the final stage of PCD during xylogenesis in zinnia in vitro. It is suggested that the enzyme is released from the vacuole after tonoplast rupture and contributes to autolysis of the cellular content of the TEs. The amino acid sequence of this enzyme was found very similar to barley endonuclease (BEN1) which participates in the breakage of nuclear DNA during the cell death of the endosperm in barley seeds (Aoyagi et al. 1998; Ito and Fukuda 2002). The autolysis is associated also with activation of RNAses (Green, 1994; Aoyagi et al. 1998; Thelen and Northcote 1989). In in vitro developing zinnia TEs a gene expression of *ZRNaseI* was found in the late stage of differentiation, whereas *ZRNaseII* appeared to be expressed in response to wounding. The same types of *ZRNase* genes were detected in differentiating xylem and in response to wound stress in zinnia plants (Ye and Droste 1996). These results demonstrated that endonucleases are implicated in the process of xylem differentiation.

Other proteolytic enzymes were also reported to accumulate in the vacuole of transdifferentiating zinnia cells and to be released after vacuolar collapse (Obara et al. 2001). Among them is thrombin-like protease (TLP) with pH optimum 5.5–6.0 that was identified in conditioned medium of zinnia cells in the stage of TE cell death. It was suggested that it participates in the collapse of the tonoplast or in the autolysis of cellular content (Yu et al. 2005). In TE-inductive zinnia culture, proteases expressing an activity against several peptidyl 4-methyl-7-coumarylamido (MCA) substrates have been found. The amount of hydrolyzed carbobenzoxy-Phe-Arg-MCA (Z-Phe-Arg-MCA), a specific substrate for cathepsin L enzyme in animal systems, was stable in freshly isolated mesophyll cells but increased in differentiation-related manner following the addition of auxin and CK. A protein with 30 kDa molecular mass, located in the vacuole, was established to be responsible for this activity and identified as cysteine endopeptidase with a pH optimum at pH 5.0 (Minami and Fukuda 1995).

Several investigations have shown that similar genes are upregulated and transcripts coding for different proteins related to SCW formation and cell death begin to accumulate at the same time suggesting that common signals may induce both PCD and SCW deposition (Fukuda (1996, 1997, 2016; Demura et al. 2002; Milioni et al. 2002; Kubo et al. 2005; Pesquet et al. 2004). An example of such coupled regulation is the papain-like Zinnia Cysteine Protease 4 (ZCP4). High abundance of transcripts of *ZCP4* is found prior to autolysis whereas the 11-bp cis-element, the tracheary-element-regulating cis-element (TERE), that is the *ZCP4* promoter and is responsible both for SCW and PCD-related genes was identified in immature TEs (Endo et al. 2009; Pesquet et al. 2004; Pyo et al. 2004, 2007). A serine protease with molecular mass of 60 kDa was identified during the progression of TE differentiation in zinnia cell culture and was suggested to contribute to cellular autolysis (Ye and Varner 1996). Groover and Jones (1999) detected a 40-kD serine protease which is secreted during SCWs synthesis and is released from the collapsed vacuole after SCWs are visually completed (Fukuda 1987). This protein was suggested as a possible coordinating factor between SCW synthesis and cell death at the end of PCD process.

Pharmacological studies revealed the participation of more proteolytic factors in the different stages of differentiation (Supplemental File S4). Among them are various specific and broad range cysteine and serine proteases and the proteasome (Minami and Fukuda 1995; Ye and Varner 1996; Woffenden et al. 1998; Groover and Jones 1999; Iakimova and Woltering 2009; Han et al. 2012; Escamez and Tuominen 2014).

Fukuda (1987) reported changes in tubulin synthesis during cell division of isolated zinnia mesophyll cells and TE differentiation. Later, in zinnia cell culture and in zinnia seedlings differentiatial gene expression has been detected for *β*-tubulin isotypes *ZeTubBl*, *ZeTubB2* and *ZeTubB3*. These genes encode for the protein tubulin which controls the orientation of newly deposited cellulose microfibrils related to the positioning of SCWs. In the cell culture the accumulation of transcripts of *ZeTubBl* and *ZeTubB3* was promoted by CK and auxin and occurred rapidly prior to cell division and SCW formation. In the seedlings *ZeTubB* transcripts were detected in the ground meristem and in procambium. Together, these findings suggested preferential expression of tubulin genes in procambium stem cells and in differentiating xylem cells (Yoshimura et al. 1996).

### Involvement of specific PCD-associated proteases

There are indications that CLPs participate in the process of transdifferentiation/PCD in zinnia cell system. Among them are the findings of Twumasi et al. (2010a) which for the first time provided experimental arguments pointing to a role of CLPs in zinnia TE cell death (Supplemental File S4). However, because in the experiments of these researchers TE formation was partially but not entirely inhibited by tetrapeptide caspase inhibitors, it was proposed that the CL enzymes might be activated in early stages of transdifferentiation, upstream of cell wall deposition or at least before visual appearance of cell wall thickenings while the cell is still alive. How CLPs may act is not clear but they might, through different still unknown mechanisms, trigger the death of TE cells in stage III of transdifferentiation and also affect SCWs synthesis and deposition in stage II. In support to this suggestion was our observation that the caspase inhibitors did have an effect on TE differentiation only if applied simultaneously with hormonal induction and not earlier or later (Iakimova and Woltering unpublished data). Han et al. (2012) reported that zinnia TE development was suppressed if the caspase-3 inhibitor Acyl-Asp-Glu-Val-l-aspartic acid aldehyde (Ac-DEVD-CHO) was introduced at time zero of culture development and not after 48 h. Together these findings show that common CLPs pathways might contribute to the early and late stages of zinnia TE development in vitro and might be engaged even during stage I when the cells acquire a competence to respond to auxin and CK.

Although the knowledge about the contribution of CLPs to TE differentiation in zinnia cell culture is still in its infancy, in other plant systems their involvement in xylogenesis has been proven (Petzold et al. 2012 and references therein). By immunohistochemical methods and immunoelectron microscopy, Hao et al. (2008) detected caspase-3-like protease localized in the cytoplasm and in the cell walls of developing TEs in *Cucurbita moschata*. During development of secondary xylem in *Populus tomentosa*, by using liquid chromatography-tandem mass spectrometry, Han et al. (2012) purified the caspase-3-like enzyme and discovered that 20S proteasome is responsible for its activity which was associated with visible cell death of xylem elements *in planta*. They found that in the presence of Ac-DEVD-CHO xylem formation in *Arabidopsis* cotyledons was suppressed, which additionally pointed to a role of caspase-3-like protease in xylem cell death. The CL enzyme VPE (a plant protease that expresses caspase-1-lke activity) has been shown to play a role in posttranslational modification of a variety of vacuolar proteins (Hatsugai et al. 2015 and references therein). In *Arabidopsis* cells differentiating in suspension, increased gene expression of *VPEα* has been determined at the early stage of differentiation (after 48 h following the hormonal induction of the cells), and a high level of transcripts was sustained during stages II and III of TE development (Kubo et al. 2005). These results suggested that VPE may contribute to vacuolar collapse during TE cell death (Turner et al. 2007 and references therein). Further, microarray analysis reported by Courtois-Moreau et al. (2009) showed up-regulation of *VPE* (homologous of *Arabidopsis* *β*-*VPE* and *γ*-*VPE*) and of Cathepsin B-like cysteine proteases (potential targets of VPE action) during secondary xylem development in *Populus*. A caspase-1-like activity, determined by capability of the enzyme to cleave the caspase-1 specific fluorogenic substrate Ac-Tyr-Val-Ala-Asp-7-Amino-4-methylcoumarin (Ac-YVAD-AMC), was detected in the xylem of *Populus tomentosa* (Han et al. 2012). Indirect evidence for possible involvement of VPE in zinnia TE differentiation in vitro came also from Twumasi et al. (2010a) who observed a reduced rate of TE generation in presence of the caspase-1 inhibitor Tyr-Val-Ala-Asp-chloromethylketone (Ac-YVAD-CMK). As VPE is implicated in almost all known forms of vacuolar PCD and is considered as one of the hallmarks of this cell death type (van Doorn and Woltering 2005, 2010; van Doorn et al. 2011), the putative role of this protease in zinnia PCD during transdifferentiation in vitro needs to be further elucidated.

Metacaspases are a class of cell death-associated proteases that are structurally related to animal caspases but have different substrate specificity (Uren et al. 2000). During the late stage of TE differentiation in *Arabidopsis* and during xylem maturation in *Populus* microarray analysis revealed upregulation of a homolog of *Arabidopsis* metacaspase 9 (*AtMC9*) (Turner et al. 2007; Courtois-Moreau et al. 2009; Bollhöner et al. 2013). In a manner resembling that of AtMC9, two papain-like cysteine proteases (PLCPs) named Xylem Cysteine Peptidase 1 (XCP1) and XCP2 were upregulated. These proteases were implicated in micro-autolysis of cellular structures before tonoplast rupture and in mega-autolysis of the entire protoplast following tonoplast breakage in differentiating TEs in *Arabidopsis* cell culture (Zhao et al. 2000; Funk et al. 2002; Avci et al. 2008). Bollhöner et al. (2013) hypothesized that *AtMC9* may regulate *XCP1*/*XCP2* but their experiments with *atmc9*-*2* and double *xcp1 xcp2* mutants showed that the metacaspase and the studied PLCPs are independently related to autolysis. The authors suggested that AtMC9 may potentially affect other papain-like proteases participating in *post*-*mortem* protoplast clearance. This presumption was substantiated by observations indicating that a cysteine protease (Tr-cp14) that is closely related to XCP1 and XCP2 accumulated in the ER and Golgi vesicles, from where it appeared to spread throughout the cell during the collapse of the central vacuole of *in planta* differentiating TEs of *Trifolium repens* (Mulisch et al. 2013). No reports about metacaspase identification in transdifferentiating cultured zinnia cells are yet available.

### DNA synthesis

In zinnia cell culture the major portion of mesophyll cells transdifferentiate into TEs without prior cell division (Church and Galston 1988; Church 1993). Initially Fukuda and Komamine (1981a) and earlier Basile et al. (1973) have suggested that cell division including whole genome replication and mitosis are not prerequisite for initiation of transdifferentiation of zinnia mesophyll cells in vitro and for transdifferentiation of pith parenchyma cells of lettuce leaf disks cultured ex vivo. However it was also found that DNA synthesis might be required and TEs can originate both from non-dividing and dividing cells (Dodds 1980; Fukuda and Komamine 1981b; Sugiyama and Komamine 1987; Kákošová et al. 2013) (Supplemental File S4).

### TE differentiation is dependent on intercellular signalling

**Fig. 4:**
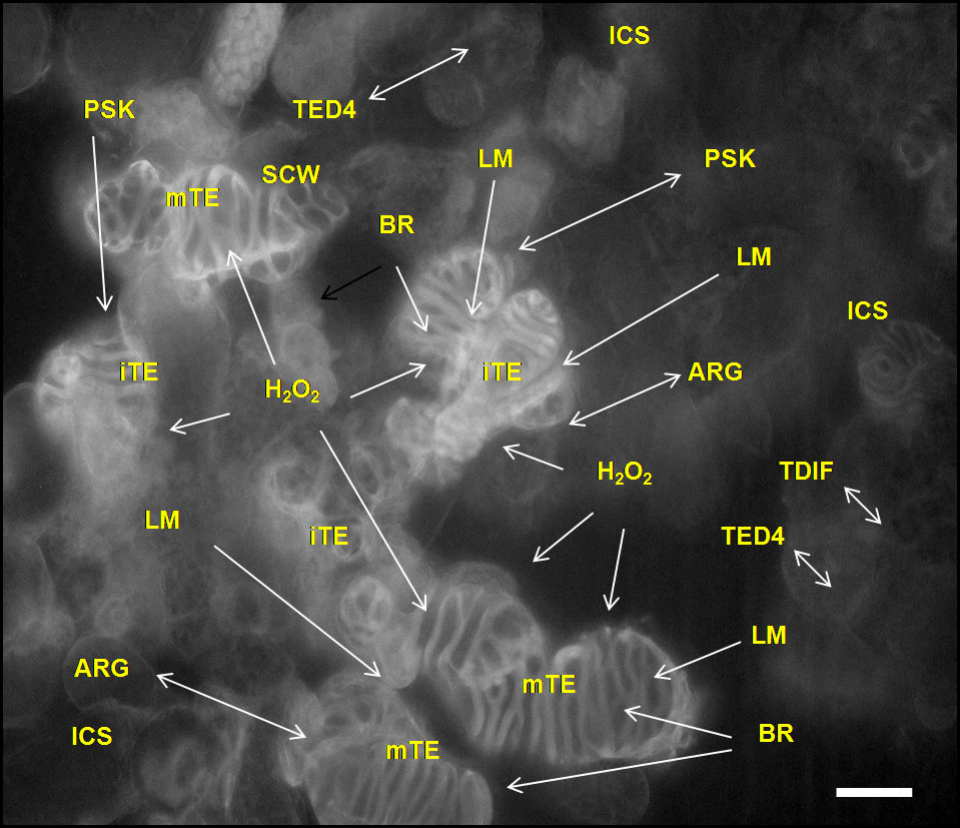
Intercellular signalling in xylogenic zinnia cell cultures. The culture contains living cells, immature (with incomplete SCWs) TEs and mature TEs (with empty cellular content and completed SCWs). The living cells release compounds into the medium that are further incorporated into the SCWs (lignin monomers and H_2_O_2_) or stimulate the transdifferentiation (ARG, PSK and BRs), or protect against the death of living cells (TED4), or suppress the differentiation of procambium and cambium cells (TDIF). A positive feedback loop is suggested for ARG and PSK in which the procambium cells produce these substances and the cells that are induced to differentiate produce more ARG and PSK that promote the transdifferentiation of not yet differentiated cells. Brassinosteroids contribute to early and late transdifferentiation stages. The SCWs of the TEs in the background image are distinguished by lignin autofluorescence (excitation/emission wavelength 470–520 nm). The image is taken under fluorescent microscope Axiovert Carl Zeiss. Arabinogalactan (ARG), brassinosteroids (BR), hydrogen peroxide (H_2_O_2_), intercellular space (ICS), immature tracheary element (iTE), mature tracheary element (mTE), lignin monomers (LM), phytosulfokine-*α* (PSK), secondary cell wall (SCW), tracheary element differentiation-related peptide (TED4), tracheary element differentiation inhibitory factor (TDIF). *Scale bar* 50 µm

The TE differentiation in the xylogenic cell cultures and *in planta* is non-autonomous process dependent on substances supplied by the living non-differentiated cells and from immature TEs. The factors involved in cell-to-cell signalling operate in complicated but well synchronised manner (Fig. 4; Supplemental File S5). The studies on the role of intercellular signalling during TE development in zinnia cell system, zinnia stems and *Arabidopsis* have revealed a messenger role of arabinogalactan (ARG)-like proteins, BRs and PSK in the control over initiation of differentiation program. Mono- and dilignols, and H_2_O_2_ produced in the living cells and transferred through extracellular space to differentiating TEs are responsible for lignification of the SCWs in the immature, maturating and mature TEs, including the process *post*-*mortem*.

A ligand-receptor pair made of the peptide Extracellular Tracheary element Differentiation Inhibitory Factor (TDIF) and TDIF RECEPTOR/PHLOEM INTERCALATED WITH XYLEM membrane protein kinase (TDR/PXY) promotes the proliferation of procambial cells and suppresses their xylem differentiation thus maintaining the balance between proliferation and differentiation. Tracheary Element Differentiation-related (TED4) peptide which is a plant non-specific lipid transfer protein performs a cell death protective function by inhibiting the proteasome mediated downstream cell death signalling. In xylogenic zinnia culture TED4 is secreted into the apoplast prior to and with the progression of morphological changes of the TEs (Ito et al. 2006). The contribution of other factors such as ATP binding cassette (ABC) transporters and Rac small guanosine-5′-triphosphatase **(**GTPase) to intercellular messaging is also established (Supplemental File S5). The involvement of various players in the intercellular signalling clearly shows that the life and death in zinnia cell cultures and the differentiation of xylem cells *in planta* is under strict control (Ogawa et al. 1997; Barceló 1998a, b; Groover et al. 1997; Endo et al. 2001; Hosokawa et al. 2001; Motose et al. 2001a, b, 2004; Yamamoto et al. 2001, 2007; Dahiya et al. 2005, 2006; Karlsson et al. 2005; Pesquet et al. 2005, 2013; Tokunaga et al. 2005; Ito et al. 2006; Fukuda et al. 2007; Avci et al. 2008; Hirakawa et al. 2010; Kobayashi et al. 2011; Kondo et al. 2011; Novo-Uzal et al. 2013; Schuetz et al. 2013; Farquharson 2014; Escamez and Tuominen 2014; Bollhöner et al. 2012, 2013; Wang et al. 2013; Ménard and Pesquet 2015; Sauter 2015; Serk et al. 2015).

### Other cell systems to study xylogenesis in vitro and novel approaches in vivo

The knowledge on the processes underlying xylem differentiation gathered from zinnia cell system has served as a basis for development of other xylogenic systems in vitro and new technologies for studies in models in vivo. Some of the recently introduced experimental systems are demonstrated also as efficient tools for research on xylogenesis and are applied in addition to or in replacement of zinnia cell system.

It is not yet fully understood which endogenous factors determine the potential of zinnia mesophyll cells to transdifferentiate in culture. Hypothetically, the transdifferentiation ability might be related to the physiological state of the young zinnia leaves, their sensitivity to wound stress that stimulates the transdifferentiation, levels of hormones, organization of cytoskeleton and other putative peculiarities. However, it was found that cell and tissue cultures derived from other species as well as isolated leaf tissues also express such differentiation capacity when supplemented with auxin and cytokinin. This suggests that the potential for transdifferentiation in vitro and ex vivo is most probably stimulated mainly by the exogenous supply with the hormones, which in turn, considerably resembles the hormonal induction of xylem cell differentiation *in planta*.

Xylogenic cell cultures and callus of angiosperm and gymnosperm species such as suspension cultures initiated from e.g. *Arabidopsis thaliana* root cells, stem callus of *Centaureae cyanus*, *Pseudostuga menziesii*, *Pinus* spp. and *Populus* spp.; callus of *Syringae vulgaris*, *Glycine max*, *Daucus carota*, *Helianthus tuberosus, Parthenocissus*, *Cucumis sativus,* and *Cupressus sempervirens* have been established (Aloni 1980; Torrey 1975; Krishnamurthy et al. 1999; Kubo et al. 2005; Oda et al. 2005; Turner et al. 2007; Avci et al. 2008; Jung et al. 2008; Pesquet et al. 2010, 2013; Bollhöner et al. 2013; Schuetz et al. 2013; Escamez and Tuominen 2014; Devillard and Walter 2014). For example, an early report informed that in presence of the right concentration of kinetin and NAA, callus from *Glycine max*, cv. Biloxi, cultured on agar, can differentiate into xylem cells (Fosket and Torrey 1969). Suspension cells and callus tissue isolated from *Centaurea cyanus* and plated in Petri dishes onto solidified cytodifferentiation promoting medium expressed xylogenic potential to differentiate into single or clustered TEs (Torrey 1975). Yamagishi et al. (2015) reported formation of TEs in suspension cultures of cells derived from the mesophyll of young green needles of the coniferous plant *Cryptomeria japonica*. The cells expressed TE differentiation capacity but transdifferentiated not when induced in the liquid culture but 7 days after have been transferred from the liquid to solidified medium.

Most of the findings obtained from zinnia cell system have been implemented into studies with xylogenic *Arabidopsis* cell cultures. These systems were proven also very efficient especially for molecular and genetic studies and in the last years to a large extent replaced the zinnia model (Fukuda 2016). Moreover, counterparts of the regulatory components in *Arabidopsis* were identified in zinnia differentiation and the PCD cascade which allowed better understanding of the processes in the zinnia cell system (described in the various parts of this review). Recent works involving novel xylogenic systems revealed more details on molecular mechanisms of xylem cell differentiation. Through microarray analysis, in a transgenic system of *Arabidopsis* suspension cells Ohashi-Ito et al. (2010) identified genes that are expressed downstream of VND6 but not downstream of SECONDARY WALL-ASSOCIATED NAC DOMAIN PROTEIN1 (SND1)—transcription factors operating as master regulators of xylem fiber cells. It was shown that whereas VND6 and SND1 regulate a number of genes in common, particularly those related to SCW formation, the genes involved in TE PCD are upregulated only by VND6. In inductive *Arabidopsis* cell suspension Kwon et al. (2010) showed that the TE cell death expresses features of autophagy including autophagy-related signalling factors such as the small guanosine-5′-triphosphate (GTP) binding protein and expression of the autophagy marker gene *ATG*. They also observed double membrane bound autophagosomes and autolysosmes. In *in planta* differentiating xylem in root cells of *Populus trichocarpa*, cytological analysis revealed symptoms of autophagy such as autophagic-like structures inside vacuole (Bagniewska-Zadworna et al. 2012, 2014). These findings provided additional evidence for autophagy-like components of the vacuolar TE PCD. By genetic analyses it was determined that the microtubule-associated proteins (MAPs) AtMAP70-1 and AtMAP70-5 are essential for localisation and patterning of SCW in the TEs formed from differentiating *Arabidopsis* suspension cells (Pesquet et al. 2010). This information further substantiated the previously established role of cytoskeleton-related factors in predefining the architecture of SCW thickening (Oda et al. 2005; Lacayo et al. 2010; Carteni et al. 2014).

Initial information for ex vivo differentiating tissues is available from studies performed in the second half of the last century. Rubery and Fosket (1969) cultured stem segments of *Coleus blumei* and found that upon induction with CK and auxin the cells at the place of wounding transdifferentiate into vessel elements. In the same system they studied the role of PAL in lignification and proposed this enzyme as a marker for xylogenesis. It has been established that explants excised from pith parenchyma cells of lettuce (*Lactuca sativa*) and cultured on MS medium with vitamins, auxin and CK form callus that further differentiate into TEs (Dalessandro and Roberts, 1971). In this xylogenic system the addition of adenosine-3′,5′monophosphate (AMP) to the culture medium stimulated the conversion of parenchyma cells into TEs thus suggesting a role of AMP in differentiation (Basile et al. 1973). Miller et al. (1984) reported that in the absence of cytokinin, the cells of cultured lettuce pith parenchyma explants transdifferentiated into xylem elements if NAA was applied together with the ethylene precursor ACC or with the ethylene releasing compound ethephon. The authors suggested that ethylene might substitute qualitatively for cytokinin and that both ethylene and auxin are required for xylem differentiation in *Lactuca.* Later, by pharmacological studies, it was shown that in xylogenic zinnia cell culture ethylene can mediate the PCD signalling during the early and later stages of TE differentiation (Pesquet and Tuominen 2011; Pesquet et al. 2013) (Supplemental File S4). This hormone was also suggested to act in cooperation with NO and PAs (Gabaldón et al. 2005; Muñiz et al. 2008; Yoshimoto et al. 2012; Milhinhos and Miguel 2013). In in vitro system of cultured isolated fruit vesicles of *Citrus limon*, Khan et al. (1986) found that at a pH lower than 3.0 the differentiation is prevented but at pH 7.0 the isolated tissue transdifferentiates in TEs and form xylem vessel-like structures. Another xylogenic system was recently reported by Negi et al. (2015). They cultured banana embryogenic cells in auxin-free MS medium, supplemented with glutamine, malt extract, biotin, sucrose and 1 µM brassinolide (medium pH 5.3), and observed that approximately 32% of the cells differentiated into xylem vessel elements. In the presence of brassinolide, TE formation was inhibited by 1 µM 2,4-D.

*Arabidopsis* Glycogen Synthase Kinase 3 (GSK3) was shown to be related to BR signalling involved in intercellular communication during xylem cell differentiation. De Rybel et al. (2009) identified bikinin, a small molecule that activates BR signalling downstream of the BR receptor. The simultaneous inhibition of seven GSK3s was found sufficient to activate BR responses in *Arabidopsis*. This discovery opened a path toward generation of mutant lines for analyzing the key regulators in the BR signalling pathway. Kondo et al. (2015) established a novel experimental system of cultured leaf disks of *Arabidopsis* in which transdifferentiation of mesophyll cells into TEs has been induced in presence of auxin, CK and bikinin. The system enabled to more profoundly study the involvement of the earlier identified in differentiating zinnia cell culture TIDF (Ito et al. 2006) and Arabidopsis CLAVATA3 (CLV3)/ENDOSPERM SURROUNDING REGION (ESR)-related (CLE) proteins in the control of procambial cells proliferation and differentiation.

Microarray analysis also revealed that in the xylogenic system of isolated leaves, the expression of genes characteristic for mesophyll and epidermal cells such as CHLOROPHYLL A/B BINDING PROTEIN 3 (CAB3), RIBULOSE BISPHOSPHATE CARBOXYLASE SMALL CHAIN 1A (RBCS1A), MERISTEM LAYER 1 (ATML1), and PROTODERMAL FACTOR 2 (PDF2) decreased suggesting that the functional identity of photosynthesizing cells was lost immediately post-induction, whereas the expression of marker genes such as MONOPTEROS (MP), TDR, ARABIDOPSIS THALIANA HOMEOBOX 8 (AtHB8), and TARGET OF MP 6 (TMO6), characteristic for procambial cells was considerably enhanced until 48 h. At 36 h the levels of xylem-specific genes such as VND6, XYLEM CYSTEINE PROTEASE 1 (XCP1), and LOB DOMAIN-CONTAINING PROTEIN 15 (LBD15) increased enormously (Kondo et al. 2015). In addition to the earlier findings (Fukuda 2004; Mähönen et al. 2006; Bishopp et al. 2011; Milhinhos and Miguel 2013), these data provided further evidence that during transdifferentiation the mesophyll cells are first converted into procambial cells and then differentiate into xylem elements (Fukuda 2016).

Metabolome and transcriptome profiling in an inducible system of protoxylem vessel elements differentiating from tobacco BY-2 suspension cells transformed with *Arabidopsis VND7*-*VP16*-*GR fusion protein* (Yamaguchi et al. 2010) revealed key metabolic regulators in the biosynthesis of SCW polymers and lignin such as fructose 6-phosphate, phosphoenolpyruvate, enzymes from shikimate pathway, the hemicellulosic polysaccharide xylan, cellulose and its precursor UDP-glucose (Ohtani et al. 2016).

Cumulatively, the current understanding of differentiation-related signalling combines the vast knowledge obtained from zinnia xylogenic cell culture and discoveries achieved in previous and novel experimental xylogenic systems. The existence of counterparts of the regulatory elements participating in xylem cell differentiation in zinnia, *Arabidopsis* and other species indicates that the basic mechanisms underlying xylogenesis/PCD in vascular plants are evolutionally conserved.

### Differentiation and PCD are tightly integrated in the process of xylogenesis

The process of PCD during xylogenesis is generally discussed from the view that the program for cell death is activated only in the terminal stage of differentiation to ensure the execution of the cellular demise. However, the studies indicate that cell death-related signalling leading to the culmination of the suicide is activated from the beginning of the differentiation cascade and both processes are under the control of similar factors. It has been shown that, e.g. in zinnia and *Arabidopsis* in vitro and *in planta* the expression of genes related to PCD and SCW formation is regulated by common transcription factors and the transcripts accumulate in similar time frame during the earlier and advanced stages of TE development (Demura et al. 2002; Milioni et al. 2002; Ohashi-Ito et al. 2010; Kubo et al. 2005; Kondo et al. 2015; Fukuda 2004, 2016). It was hypothesised that VND6 and VND7, directly or indirectly regulate genes that contain the tracheary element-regulating *cis*-element (TERE) sequence to induce PCD and SCW formation in a coordinated manner (Ohashi-Ito et al. 2010). In transdifferentiating zinnia cells the studies of Twumasi et al. (2010a) suggested that PCD-associated CLPs may be activated before the visual appearance of SCWs. In stages preceding the cell death execution death proteases were identified also in *in planta* developing xylem elements of *Populus* and *Cucurbita* (Courtois-Moreau et al. 2009; Hao et al. 2008; Han et al. 2012). Gene expression of a CLP was detected in the early differentiation stages in inductive culture of *Arabidopsis* suspension cells and in *in planta* differentiating xylem of this species (Kubo et al. 2005; Courtois-Moreau et al. 2009). Moreover, exogenous administration of cell death inducers or inhibitors before or at the beginning of transdifferentiation in zinnia cell culture was shown to affect the final stage of cell death, thus promoting or suppressing the formation of completed TEs. Van Durme and Nowack (2016) presented a model of the mechanisms controlling the different phases of differentiation-induced PCD showing that the differentiating cells need first to acquire competence to undergo PCD. This includes accumulation of lytic enzymes in the cellular compartments of the young cells and further induction of signalling cascade involving messengers such as Ca^2+^, ROS and others to trigger the initiation of cell death execution. Together the findings show that differentiation and PCD are two tightly cooperating processes in the developmental program of xylogenesis. Taking this in mind, we suggest defining the entire process of xylogenesis as a form of vacuolar PCD involving differentiation events and culminating in cell death execution. A similar reasoning has been put forward to redefine the process of leaf senescence. Although leaf senescence has been viewed by several authors as a process consisting of different phases i.e. a senescence phase and a PCD phase, there are many arguments to view the whole process as a form of vacuolar PCD (van Doorn and Woltering, 2004). Once the decision to die has been made, the process follows a program in which first the cell content is degraded and redistributed to other (non-senescing) organs and finally the cell dies following disintegration of the vacuolar membrane.

### Perspectives for practical application of the fundamental findings

Implementation of the theoretical knowledge gained from the studies on xylogenesis into practical applications is a challenging goal. The dimensions of xylem vessels *in planta* vary between the genotypes, developmental stage and the positioning of xylem bundles in roots, stems and leaves and are determined by the number and size of the TEs. The TE size can be influenced also by growth conditions and stresses including microbial infection, salinity, drought and others. Misregulation of xylogenesis may cause malformations of TE anatomy resulting in defective structure of xylem conduits, deformation of root, stem and leaf organs and finally in imbalanced water flow. Studies have shown that the TE size and timing of formation in zinnia in vitro and in vivo can be manipulated by exogenous agents affecting various processes including PCD and the control over extracellular osmolarity. In addition TE dimensions can be manipulated by light condition (Supplemental File S6) (Fukuda 1996, 1997, 2000, 2004; Kenrick and Crane^1997^; McCann 1997; Groover and Jones 1999; Lee et al. 2000; Roberts and McCann 2000; Sachs 2000; van Ieperen et al. 2001, 2002; Kozela and Regan 2003; Tyree 2003; Lee and Roberts 2004; Turner et al. 2007; Muñiz et al. 2008; Brodribb 2009; Bollhöner et al. 2012; Schuetz et al. 2013).

A possibility for practical decisions is to modulate the xylem differentiation in intact plants by optimizing the cultivation conditions and eventually by supplementary treatments with substances affecting the process. Modification of the hydraulic properties of water conducting system can enhance the tolerance to water stress by ensuring better capacity for recovery after drought conditions (van Ieperen et al. 2001, 2002; Fukuda, 2004; Twumasi et al. 2005, 2008; 2010b). The number and diameter of xylem tubes in the base of cut flowers may influence the water uptake during the post-harvest performance (van Doorn 1997; van Ieperen et al. 2001, 2002). An appropriate biotechnological engineering of xylem architecture can result in extension of the shelf life (Twumasi et al. 2005, 2010b).

Increasing the production of xylem tissue in economically important trees through corrections of the genetic and metabolic program for lignification and xylem vessel cell death is another target for translational research (Tokunaga et al. 2006; Escamez and Tuominen 2014). The availability of bioengineered trees with increased production of xylem tissue that is the main constituent of the bulk wood is important for wood and paper industry as a potential source of biofuel and biomaterials (Tokunaga et al. 2006; Escamez and Tuominen 2014; Furtado et al. 2014). Moreover, cellulose and lignin in SCWs of differentiated xylem cells are thought to play a role in the natural carbon cycle, which makes the studies on xylogenesis valuable also from environmental point of view (Boudet et al. 1995; Fukuda 2004).

## Concluding remarks

Physiological and molecular studies support the view that xylogenesis is a type of vacuolar PCD. The research in xylogenic model systems show that TE differentiation proceeds through a complicated but precisely orchestrated series of regulatory and signalling pathways which remarkably resembles the processes of xylem differentiation *in planta*.

Until recently, the xylogenic zinnia cell culture was used as a basic experimental tool for investigating the different steps in the process. The knowledge collected through this cell system has enabled many breakthroughs in xylogenesis research and has provided a solid background for investigations in other models in vitro and in vivo. However, due to some limitations regarding the molecular markers and a lack of appropriate mutant cell lines, the zinnia model has to some extent been replaced by xylogenic suspension cultures of *Arabidopsis* and very recently, with novel in vivo experimental systems and approaches to elucidate the signalling pathways and genetic control over differentiation (Fukuda 2016). Although the advanced studies provide more and more clarity on the processes, there are still questions on the regulation of xylogenesis that remain to be answered. These require, among others, more profound dissection of the molecular components involved in auxin and CK perception and identification of differentiation related molecular targets of NO, ethylene, PAs, PSK, BRs and GA3, and a more thorough exploration of the interaction of the metabolic pathways of these and other hormones with CK and auxin; their contribution to the initiation of differentiation and PCD signalling cascade and to the entry into the phase of final cellular demise. The participation of specific PCD-related proteases in the different stages of differentiation is still far from well understood. Elucidation of the mode of action of CL enzymes not only in TE cell death execution but also during the earlier stages in the progression of (trans)differentiation is an interesting challenge. Determination of the possible involvement of VPE which is commonly recognised as an important player in vacuolar type of cell death will bring more clarity on the control over TE PCD. The expansion of investigations toward such and other relevant directions in xylogenesis will provide further insight into the complex regulatory steps in this developmental PCD event of utmost importance for the biological identity and the physiological functions of the vascular plants.

### *Author contribution statement*

ETI conceived the idea, collected and discussed the literature, drafted and revised the manuscript. EJW contributed to manuscript structuring, discussion and revision. Both authors have read and agreed on the manuscript.

## Electronic supplementary material

Below is the link to the electronic supplementary material.

Supplementary material 1 (DOCX 38 kb)

Supplementary material 2 (DOCX 43 kb)

Supplementary material 3 (DOCX 51 kb)

Supplementary material 4 (DOCX 54 kb)

Supplementary material 5 (DOCX 47 kb)

Supplementary material 6 (DOCX 21 kb)

## Abbreviations

2,4-D: 2,4 Dichlorophenoxyacetic acid
ABA: *cis*-Abscisic acid
ACC: 1-Aminocyclopropane-1-carboxylic acid
Ac-DEVD-CHO: Acyl-Asp-Glu-Val-l-aspartic acid aldehyde
ACO: ACC oxidase
ACS: ACC synthase
Ac-YVAD-CMK: Tyr-Val-Ala-Asp-chloromethylketone
AIB: 1-Aminoisobutyric acid
ARG: Arabinogalactan
AVG: Aminoethoxyvinylglycine
BA: *N* ^6^-benzyladenine
BR: Brassinosteroid
CaM: Calmodulin
CFW: Calcofluor white
CK: Cytokinin
CLPs: Caspase-like proteases
CLSM: Confocal laser scanning microscopy
CO: Cell osmolarity
DAF-DA: 4,5-Diaminofluorescein-2 diacetate
DCB: 2,6,-dichlorobenzonitrile
DCFH-DA: 2′-7′Dichlorofluorescein diacetate
E64: l-transepoxysuccinyl-leucylamido-[4-guanidino]butane
EC: Electrical conductivity
EO: Extracellular osmolarity
ER: Endoplasmic reticulum
FDA: Fluorescein diacetate
FDA: Fluorescein diacetate
GA3: Gibberellin
GGMOs: Galactoglucomannan oligosaccharides
GSH: Glutathione
GSSG: Glutathione disulfide
HFCA: 9-Hydroxyfluorene-9-carboxylic acid
HR: Hypersensitive response
JA: Jasmonic acid
LAC: *Clasto*-lactacystin b-lactone
LI: Light intensity
LLL: Carbobenzoxy-leucinyl-leucinyl-leucinal
LO: Leaf osmolarity
MX-like: Metaxilem-like
NAA: α-Naphthalene-acetic acid
NO: Nitric oxide
NPA: 1-*N*-naphthylphthalamic acid
PAL: Phenylalanine ammonia-lyase
PAs: Polyamines
PCD: Programmed cell death
PGRs: Plant growth regulators
PI: Propidium iodide
PLCP: Papain-like cysteine protease
PSK: Phytosulfokine-*α*
PTIO: 2-Phenyl-4,4,5,5-tetramethyl imidazoline-1-oxyl-3-oxide
PX-like: Protoxylem-like
R-like: Reticulate-like
RNS: Reactive nitrogen species
ROS: Reactive oxygen species
SA: Salicylic acid
SCW: Secondary cell wall
SNAP: *S*-nitroso-*N*-acetyl-penicillamine
SNP: Nitroprusside
TDIF: Differentiation inhibitory factor
TE: Tracheary element
TED: Tracheary element differentiation-related peptide
TEM: Transmission electron microscopy
TERE: Tracheary-element-regulating *cis*-element
TFP: Trifluoperazine
TIBA: 2,3,5-Triiodobenzonic acid
TUNEL: Terminal deoxynucleotidyl transferase mediated dUTP nick end labeling
VPE: Vacuolar processing enzyme
W-7: *N*-(6-Aminohexyl)-5-chloro-1-naphthalenesulfonamide hydrochloride
XCP: Xylem cysteine peptidase
Z-Asp-CH2-DCB: Benzyoxycarbonyl-Asp-2,6-dichlorobenzoyloxymethylketone
ZCP4: Zinnia cysteine protease 4
ZEN: Zinnia endonuclease
