# Supplementary material for: Xylogenesis in zinnia (Zinnia elegans) cell cultures: unravelling the regulatory steps in a complex developmental programmed cell death event
Source: Planta. 2017 Feb 13;245(4):681–705. doi: 10.1007/s00425-017-2656-1 (PMC5357506; doi:10.1007/s00425-017-2656-1)
Supplement: Supplementary file 1 — Supplementary material 1 (DOCX 38 kb) [file 425_2017_2656_MOESM1_ESM.docx]

Title: **Xylogenesis in zinnia (*Zinnia elegans*) cell cultures: unravelling the regulatory steps in a complex developmental programmed cell death event**

Journal: Planta

Authors: Elena T. Iakimova^1^, Ernst J. Woltering^2,3^

^1^Institute of Ornamental Plants, Sofia, Bulgaria, ^2^Wageningen University and Research, Food & Biobased Research and ^3^Wageningen University, Horticulture and Product Physiology, Wageningen, The Netherlands

Corresponding author: Ernst J. Woltering

e-mail: ernst.woltering@wur.nl

**Plant PCD categories and terminology**

The earliest reports on PCD occurrence come from the beginning of 20^th^ century. Stakman (1915) described suicidal death of plant cells of oat, rye, wheat and barley during incompatible interactions with fungal phytopathogens belonging to species *Puccinia*. The author found that the cell death occurred rapidly in limited number of cells at the place of inoculation with the fungus, before the actual penetration of the fungal hyphae into the cell. The cell death was featured with chloroplasts destruction and disappearance, nucleus disintegration and collapse of the protoplast. The process was related to the resistance of the species to the fungus and was defined as “hypersensitiveness”. Similar cell death symptoms have been observed in the mesophyll cells of wheat leaves, inoculated with *Puccinia* *graminis tritici* (Allen 1923). The actual term PCD has been introduced by Williams (1961) and Lockshin and Williams (1964) to describe the hormonal regulated death of cells in muscle tissues during metamorphosis of the insect silkworm.

In human and monkey cells PCD was first reported and termed apoptosis by Kerr et al. (1972). Further, PCD was described in all eukaryotic organisms. Kerr et al*.* (1972) detected apoptotic cellular changes featured by cytoplasm shrinkage, nucleus degradation and formation of membrane confined apoptotic bodies containing the remnants of degraded cellular content. In animal systems, the apoptotic bodies are removed through phagocytosis by adjacent cells - a process not existing in plants. However, counterparts of a number of other animal cell death features and pro- and anti-apoptotic factors have been observed in plant cells.

During the last decade the morphological categorization of plant PCD has undergone dynamic reconsideration. The Nomenclature Committee on Cell Death 2009 recommended general formulations of animal cell death as apoptosis, autophagic cell death and necrotic (non-lysosomal) cell death distinguished by morphological criteria and biochemical determinants (Kroemer et al. 2009). In analogy to animal cell death, for some time PCD events in plants have been termed apoptosis or apoptosis-like. Later, several authors have tried to categorize plant cell death types. It was argued that true apoptosis could not exist in plants as it is unlikely that in plants the content of the dying cell is “eaten” by other cells. Autophagic cell death could apply to cell death in many developmental processes as these are accompanied by a loss of cytoplasm and cytoplasmic organelles and a growing vacuole (van Doorn and Woltering 2010). However, real autophagosomes (double membrane vesicles typical for animal autophagy) had not been convincingly detected in plants. Apoptosis-like terminology was also thought to be inadequate as it was based on features that are present in cells dying in different ways. Therefore, the authors introduced the terms developmental cell death and pathogenic cell death (van Doorn and Woltering 2005, 2010). It has been assumed that developmental cell death is a relatively slow cell death process in developmental (or abiotic stress related) events (van Doorn and Woltering 2004, 2005; Love et al. 2008). This type of cell death shows similarities to animal autophagic cell death. Pathogenic cell death is considered a relatively rapid process occurring in plant-pathogen interactions and in response to chemical treatments. This form of cell death resembles animal non-lysosomal cell death as generally the cellular content is not degraded. The cells are suggested to kill themselves by inhibiting some major biosynthetic pathway, by destabilizing their membranes, or in other unknown ways (van Doorn and Woltering 2005, 2010). It has been generally assumed that depending on the inducing stimuli and rapidity with which cell death is required, different PCD forms may operate in regulatory overlap and can simultaneously occur in the same cells or in different cells of the plant tissue (van Doorn and Woltering 2005, 2010; Love et al. 2008).

Further, a larger group of plant cell death scientists published a consensus paper suggesting a new terminology (van Doorn et al*.* 2011). They defined two major classes of programmed death: vacuolar cell death and necrosis. The vacuolar cell death is thought to resemble autophagic cell death in animal systems and is usually observed in many developmental cell death events. Necrosis is the type of cell death to occur generally in response to chemical or environmental stresses and is accompanied by rapid protoplast shrinkage. Mixed cell death type is often observed at the hypersensitive response (HR) in plant pathogen interactions: a rapid local cell death occurring in a small number of cells at the primary site of pathogen attack aimed at restricting pathogen infection (Stakman 2015; Heath 2000; Mur et al. 2008). It was assumed by the authors (van Doorn et al. 2011) that the above would become the standard or at least the basis for cell death classification. However, the accuracy of term necrosis as a single plant PCD type, as suggested by van Doorn et al. (2011), was next criticized by Reape and McCabe (2013). These authors argue that condensed protoplast morphology may be caused by different mechanisms: an active cell death-associated cellular retraction (as in animal apoptosis) or simple rupture of the plasma membrane. In similarity to Van Doorn and Woltering (2005) they noted that at stressful conditions either apoptosis-like cell death (cell death-associated protoplast retraction) or necrosis (cell membrane rupture-associated retraction) is expressed depending on the severity of the stress stimulus. Therefore the authors suggested distinguishing between apoptosis-like PCD and necrosis for cell death that is associated with protoplast shrinkage.

The debates on plant PCD deepen the understanding of the diversity of plant cell deaths and expand the recognition of the differences and similarities between animal and plant PCD. However, they also cause confusion in the terminology and in the definition of PCD types not covered by the recommended major categories, which especially applies to the various forms of intermediate cell death morphology. Moreover, the above mentioned classifications refer mainly to higher plants and poorly address the PCD occurrence in, for example, unicellular algae and lower plants such as mosses and lichens. Particularly in unicells, various mixed features of necrotic and vacuolar cell deaths are described (Yordanova et al*.* 2013 and references therein; Bidle 2015). The progressing dispute soundly demonstrates that the knowledge on plant PCD is incomplete and for more precise discrimination of PCD classes, additional biochemical and molecular criteria should be considered.

**References**

Allen RA (1923) Cytological studies of infection of Baart, Kanred and Mindum wheats by *Puccinia graminis tritici* forms III and XIX. J Agr Res 26(12):571-604

# Bidle KD (2015) The molecular ecophysiology of programmed cell death in marine phytoplankton. Annu Rev Mar Sci 7:341-375. doi: 10.1146/annurev-marine-010213-135014

# Heath MC (2000) Hypersensitive response-related death. Plant Mol Biol 44:323-334. doi: 10.1007/978-94-010-0934-8_6

Kerr JF, Wyllie AH, Currie AR (1972) Apoptosis: a basic biological phenomenon with wide ranging implications in tissue kinetics. British J Cancer 26:239-257.

Kroemer G, Galluzzi L, Vandenabeele P, et al (2009) Classification of cell death. Recommendations of the Nomenclature Committee on Cell Death 2009. Cell Death Differ 16:3-11. doi:10.1038/cdd.2008.150

Lockshin RA, Williams CM (1964) Programmed cell death-II. Endocrine potentiation of the breakdown of the intersegmental muscles of silkmoths. J Ins Physiol 10:643-649. [doi:10.1016/0022-1910(64)90034-4](http://dx.doi.org/10.1016/0022-1910(64)90034-4)

Love AJ, Milner JJ, Sadanandom A (2008) Timing is everything: regulatory overlap in plant cell death. Trends Plant Sci 13:589-595. [doi: 10.1016/j.tplants.2008.08.006](http://dx.doi.org/10.1016/j.tplants.2008.08.006)

Mur LAJ, Kenton P, Lloyd AJ, Ougham H, Prats E (2008) The hypersensitive response; the centenary is upon us but how much do we know? J Exp Bot 59:501-520. doi: 10.1093/jxb/erm239

# Reape TJ, McCabe PF (2013) Commentary: The cellular condensation of dying plant cells: Programmed retraction or necrotic collapse? Plant Sci [207](http://www.sciencedirect.com/science/journal/01689452/207/supp/C):135-139.

Stakman EC (1915) Relation between *Puccina graminis* and plants highly resistant to its attack. J Agr Res 4:193-199.

van Doorn WG, Woltering EJ (2004) Senescence and programmed cell death: substance or semantics? J Exp Bot 55:2147-2153. doi: 10.1093/jxb/erh264

van Doorn WG, Woltering EJ (2005) Many ways to exit? Cell death categories in plants. Trends Plant Sci 10:117-122. [doi:10.1016/j.tplants.2005.01.006](http://dx.doi.org/10.1016/j.tplants.2005.01.006)

van Doorn WG, Woltering EJ (2010) What about the role of autophagy in PCD? Trends Plant Sci 15:361-362. doi: [10.1016/j.tplants.2010.04.009](http://dx.doi.org/10.1016/j.tplants.2010.04.009)

van Doorn, Beers EP, Dangl JL, et al (2011) Morphological classification of plant cell deaths. Cell Death Differ 18:1241-1246. doi: 10.1038/cdd.2011.36

Williams CM (1961) The juvenile hormone. II. Its role in the endocrine control of molting, pupation, and adult development in the *Cecropia* silkworm. Biol Bull Woods Hole 121:572-585.

Yordanova ZP, Woltering EJ, Kapchina-Toteva VM, Iakimova ET (2013) Mastoparan-induced programmed cell death in the unicellular alga *Chlamydomonas reinhardtii.* Ann Bot 111:191-205. doi: 10.1093/aob/mcs264
