# Supplementary material for: Xylogenesis in zinnia (Zinnia elegans) cell cultures: unravelling the regulatory steps in a complex developmental programmed cell death event
Source: Planta. 2017 Feb 13;245(4):681–705. doi: 10.1007/s00425-017-2656-1 (PMC5357506; doi:10.1007/s00425-017-2656-1)
Supplement: Supplementary file 2 — Supplementary material 2 (DOCX 43 kb) [file 425_2017_2656_MOESM2_ESM.docx]

Title: **Xylogenesis in zinnia (*Zinnia elegans*) cell cultures: unravelling the regulatory steps in a complex developmental programmed cell death event**

Journal: Planta

Authors: Elena T. Iakimova^1^, Ernst J. Woltering^2,3^

^1^Institute of Ornamental Plants, Sofia, Bulgaria, ^2^Wageningen University and Research, Food & Biobased Research and ^3^Wageningen University, Horticulture and Product Physiology, Wageningen, The Netherlands

Corresponding author: Ernst J. Woltering

e-mail: ernst.woltering@wur.nl

**Summary of procedure for establishment of xylogenic zinnia cell culture with a focus on the critical steps**

The transdifferentiation ability of cultured zinnia mesophyll cells is related to the physiological state of the young zinnia leaves, their sensitivity to wound stress that stimulates the transdifferentiation, endogenous levels of hormones, organization of the cytoskeleton and other perhaps not yet known factors. In addition to these peculiarities, the realization of the xylogenic capacity in zinnia culture is strongly dependent on the experimental conditions.

Basically, seedlings of zinnia (*Zinnia elegans*), cultivars such as Canary Birds, Envy, Purple Prince, Peter Pan or others that have been shown to express a sufficient differentiation rate, are raised in peat-based commercial potting compost (e.g. 85 % peat, 15 % clay), vermiculite or another inert substrate under controlled environment of 16 h day photoperiod, light intensity 200 μmol photons m^-2^ s^-1^ irradiance, day/night temperature 25/20 ^o^C and relative air humidity lower than 70 % (Fukuda and Komamine 1980; Twumasi et al. 2009, 2010a, b). Other comparable growing conditions might also be appropriate. Depending on the purpose of study, recalcitrant zinnia cultivars with lower differentiation capacity, such as cv. Palette d’Artiste might also be used (Weir et al. 2005).

The mesophyll cells are isolated from whole young leaves (preferably first true leaves of 14 day old seedlings). Cells from the older leaves may transdifferentiate at a lower rate or lack transdifferentiation potential (Fukuda and Komamine 1980). To separate the single mesophyll cells, surface-sterilized leaves are gently macerated in a culture medium at sterile conditions. Gentle homogenization of leaf tissue is important due to necessity for obtaining sufficient amount of living cells in the culture (see below). The medium composition basically consists of macro- and microelements, vitamins and their precursors, sucrose and D-mannitol (Fukuda and Komamine, 1980). An essential nutrient element suggested to influence the differentiation is nitrogen, of which the source and amount in the *in vitro* culture need to be properly balanced. A combination of 20 mM potassium nitrate with 1 mM ammonium chloride has been recommended (Fukuda and Komamine 1980). Roberts et al. (1992) developed a simplified zinnia culture medium in which the concentrations of inorganic salts were reduced and from the organic components only thiamine, nicotinic acid, auxin, CK, sucrose and mannitol were present. Due to elimination of ingredients interacting with calcium signalling such as myo-inositol and EDTA and the reduced amount of Mg^2+^ and Mn^2+^ this medium was proposed as suitable for studies on the role of Ca^2+^ in TE differentiation.

In the following step, the homogenate of macerated leaf tissue is filtered through a sterile 50 μm mesh and centrifuged at 150 g for 90 s and the pellet re-suspended in the medium. The obtained suspension culture is maintained in the dark at 26 ^o^C, on orbital shaker at 80-100 rpm (Twumasi et al. 2009, 2010a, b). Because only the living mesophyll cells can transdifferentiate, their vitality in the culture is a critical factor. For achieving transdifferentiation, at least 40-50 % of the mesophyll cells should be vital. The wound stress at the isolation of leaf parenchyma cells is a prerequisite for their dedifferentiation. Mechanically damaged cells are suggested to release wound signals involved in the initiation of transdifferentiation (Fukuda 1996; Matsubayashi et al. 1999; Motose et al. 2004, 2009). However, it should be noted that severe injury at isolation may cause cell death thus diminishing the amount of living cells capable to transdifferentiate. Another condition for transdifferentiation is the cell density in the population which is required to be at least 10^5^ cells mL^-1^ or higher. At cell concentration lower than 10^4^ cells mL^-1^ the death of mesophyll cells is highly increased resulting in suppressed differentiation rate (Fukuda and Komamine 1980; Matsubayashi et al. 1999; Motose et al. 2001a, b; Turner et al. 2007). Using a proper isolation procedure the freshly collected culture is expected to contain at least 80 % viable cells.

For induction of TE differentiation, 24 – 48 h after isolation the cell culture must be supplemented with auxin and CK, the ratio and concentration of which are critical factors (Fukuda and Kommamine 1980; Church 1993). Freshly isolated culture is unstable and the capacity of mesophyll cells to transdifferentiate after induction with hormones is sustained until 48 h post-isolation. Initially Fukuda and Komamine (1980) recommended 10:1 ratio of CK : auxin (1 mg L^-1^ N^6^-benzyladenine (BA) and 0.1 mg L^-1^ α- naphthaleneacetic acid (NAA) or 2,4 dichlorophenoxyacetic acid (2.4-D) and obtained about 30 % differentiated TEs. In later experiments 1:1 ratio of the hormones (e.g. 1 mg L^-1^ BA and 1 mg L^-1^ NAA) was found appropriate for yielding 60-74 % TEs (Church 1993; Fukuda 1996, Twumasi et al. 2009). Similar amount of TEs has been recorded at equal concentrations of both hormones (0.1 mg L^-1^ or 0.2 mg L^-1^) or 2:1 ratio of CK : auxin such as 0.2 mg L^-1^ BA and 0.1 mg L^-1^ NAA (Ye and Varner 1996; Yamamoto et al. 2001; Muñiz et al. 2008). After the induction, the culture is maintained at the same conditions as the freshly prepared culture. Immature TEs usually appear approximately within 72 h and the first fully differentiated cells may be observed 96 - 120 h post induction***.***

Noteworthy is that TE differentiation might vary by rate, might not always proceed perfectly synchronous and, never 100 % of the cells transdifferentiate. Among the possible reasons, differences in cellular (CO) and extracellular osmolarity (EO) have been suggested (Lee and Roberts 2004). The cell osmolarity affects the expansion of mesophyll cells which is inversely correlated to transdifferentiation. According to Lee and Roberts (2004) EO in the range of 200 - 400 mOsm might cause suppression of cell expansion and stimulate TE differentiation. However, osmolarity above 300 mOsms might cause plasmolysis of the mesophyll cells resulting in decline of the number of generated TEs. The CO in the population of isolated mesophyll cells might range between 250 and 600 mOsm which might at least in part explain the variations in the synchrony of TE differentiation: repression of the process at lower CO (due to promotion of cell expansion) and at higher CO (due to plasmolysis). To this regard, in the experimental conditions, the EO needs to be well adjusted by osmolytes such as mannitol and sucrose.

Another factor affecting the transdifferentiation is the pH of culture medium. In their original protocol Fukuda and Komamine (1980) recommended pH 5.5. Roberts and Haiglier (1994) found that pH influences the growth rate, size, shape, SCW patterning and the timing of TE formation. They established that pH of the medium changes naturally during the time course of TE development. In their experiments zinnia TEs were detected between 48 and 60 h post-induction. The initial pH of 5.5 decreased below 5.0 prior to the onset of TE differentiation and was sustained at 4.8 - 4.9 for the whole period when new TEs continued to appear. When the TE formation ceased, pH increased to 5.6 at 79 - 80 h and thereafter decreased again remaining at approximately 5.5 until 120 h of culturing. In media with pH set at values between 4.5 and 7.0 through buffering with 20 mM 2-(*N*-morpholino)ethanesulfonic acid (MES), the percentage of formed TEs in pH range 4.5 - 6.0 did not substantially differ from the unbuferred culture but at pH 4.5, 5.5 and 6.0 a delay in the timing of TE appearance was observed. At pH 7.0 the differentiation was almost totally prevented. At pH 5.0 the timing of TE differentiation and the amount of TEs was not changed as compared to non-buffered culture which suggested that pH 5.0 might be an optimum. These results indicated that the process of transdifferentiation can be controlled by manipulating the extracellular pH. For example, Roberts and Haigler (1994) established that through regulation of extracellular pH, the portion of metaxylem (MX)-like TEs with reticulate SCW thickenings can be increased by prolonging the phase for cell expansion before the initiation of transdifferentiation process. Large MX-like TEs appeared in buffered culture with pH range 5.5 - 6.0. The described effects of osmolarity and pH suggest a link between the regulatory mechanisms controlling cell expansion and TE differentiation. Interestingly, in non-buffered cultures two stages of differentiation have been observed: synchronous TE formation during the first one and asynchronous during the second (Falconer and Seagull 1988; Roberts and Haigler 1994). The reason behind these variations is unclear but, it was assumed that the early acidification of the medium might facilitate the auxin uptake thus stimulating synchronous TE differentiation to the first peak whereas the further increase of pH might cause a disturbance of synchrony through inhibiting the differentiation in a part of the TEs (Roberts and Haigler 1994 and references therein). The ‘two-waves’ pattern of TE production has been reported in the work of Twumasi et al. (2010a) and was discussed in relation to eventual contribution of PCD specific proteases. This suggestion arose from experiments showing that the inhibition of such enzymes leads to elimination of the second peak of TE generation and after the first ‘wave’ until 120 h post-induction the TE formation continued to increase continuously but in a slower pace.

The transdifferentiation might be suppressed if the culture is contaminated. We have observed that in a case of mild fungal and/or bacterial infection (microbes not-identified) no substantial increase of the death of non-differentiated mesophyll cells occurred but the TE development was totally prevented (Iakimova and Woltering unpublished data). A simple explanation might be that the pathogens consume components of the culture medium thus depleting the plant cells of nutrients and/or hormones necessary for transdifferentiation. However, the malnutrition of the cells would be expected to cause elevation of cell mortality which was not the case. Therefore, we suggested that another more likely reason for abolishment of transdifferentiation could be that the microbial cells might produce compounds that alter the plant cell metabolism leading to inhibition of the acquisition of competence for responding to CK and auxin, and in such a way affecting the transition toward transdifferentiation. In support to this assumption is the finding of Takeuchi et al*.* (2013) who reported that a fungal elicitor derived from *Botrytis cinerea*, and chitosan (a N-deacetylated derivative of chitin which is a component of fungal cell walls) inhibited the transition of dedifferentiated cells into xylem cell precursors and the conversion of the latter to immature TEs. Hypothetically, a release from plant cells of substances serving for defence against pathogens but negatively influencing the transdifferentiation is also possible. Investigations on plant-microbe interactions during xylogenesis *in vitro* may reveal signals involved in biotic stress-induced disturbance of xylem formation *in planta*.

**References**

Church DL (1993) Tracheary element differentiation in Zinnia mesophyll cell cultures. Plant Growth Regul 12:179-188. doi:10.1007/BF00027197

# Falconer MM, Seagull RW (1988) Xylogenesis in tissue culture III: Continuing wall deposition during tracheary element development. Protoplasma 144:10-16. doi: 10.1007/BF01320275

Fukuda H (1996) Xylogenesis: initiation, progression and cell death. Annu Rev Plant Physiol Plant Mol Biol 47: 299-325. DOI: 10.1146/annurev.arplant.47.1.299

Fukuda H, Komamine A (1980) Establishment of an experimental system for the study of tracheary element differentiation from single cells isolated from the mesophyll of *Zinnia elegans.* Plant Physiol 65:57-60. doi: [​10.​1104/​pp.​65.​1.​57](http://dx.doi.org/10.1104/pp.65.1.57)

Lee S, Roberts AW (2004) Tracheary element differentiation is correlated with inhibition of cell expansion in xylogenic mesophyll suspension cultures. Plant Physiol Bioch 42:43-48. [doi:10.1016/j.plaphy.2003.10.05](http://dx.doi.org/10.1016/j.plaphy.2003.10.005)

Matsubayashi Y, Takagi L, Omura N, Morita A, Sakagami Y (1999) The endogenous sulfated pentapeptide phytosulfokine-α stimulates tracheary element differentiation of isolated mesophyll cells of Zinnia. Plant Physiol 120:1043-1048. doi: [http:/​/​dx.​doi.​org/​10.​1104/​pp.​120.​4.​1043](http://dx.doi.org/10.1104/pp.120.4.1043)

Motose H, Fukuda H, Sugiyama M (2001a) Involvement of local intercellular communication in the differentiation of zinnia mesophyll cells into tracheary elements. Planta 213:121-131. doi: 10.1007/s004250000482

Motose H, Sugiyama M, Fukuda H (2001b) Cell-cell interactions during vascular development. J Plant Res 114:473-481. doi: 10.1007/pl00014014

Motose H, Sugiyama M, Fukuda H (2004) A proteoglycan mediates inductive interaction during vascular development. Nature 429:873-878. doi:10.1038/nature02613

Motose H, Iwamoto K, Endo S, et al (2009) Involvement of phytosulfokine in the attenuation of stress response during the transdifferentiation of *Zinnia* mesophyll cells into tracheary elements. Plant Physiol 150:437-447. doi: [10.​1104/​pp.​109.​135954](http://dx.doi.org/10.1104/pp.109.135954)

Muñiz L, Minguet EG, Singh SK*,* et al (2008) ACAULIS5 controls *Arabidopsis* xylem specification through the prevention of premature cell death. Development 135:2573-2582. doi: 10.1242/dev.019349

Roberts AW, Haigher CH (1994) Cell expansion and tracheary element differentiation are regulated by extracellular pH in mesophyll cultures of *Zinnia elegans* L. Plant Physiol 105:699-706. doi: [​10.​1104/​pp.​105.​2.​699](http://dx.doi.org/10.1104/pp.105.2.699)

Roberts AW, Koonce LT, Haigler CH (1992) A simplified medium for *in vitro* tracheary element differentiation in mesophyll suspension cultures from *Zinnia elegans* L. Plant Cell Tiss Org 28:27-35. doi: 10.1007/bf00039912

Takeuchi C, Nagatani K, Sato Y (2013) Chitosan and a fungal elicitor inhibit tracheary element differentiation and promote accumulation of stress lignin-like substance in *Zinnia elegans* xylogenic culture. J Plant Res 126:811-821. doi: 10.1007/s10265-013-0568-0

Turner S, Gallois P, Brown D (2007) Tracheary element differentiation. Annu Rev Plant Biol 58:407-433. doi: 10.1146/annurev.arplant.57.032905.105236

Twumasi P, Schel JHN, van Ieperen W, Woltering EJ, Van Kooten O, Emons AMC (2009) Establishing *in vitro Zinnia elegans* cell suspension cultures with high tracheary element differentiation. Cell Biol Int 33:523-533. [doi: 10.1016/j.cellbi.2009.01.019](http://dx.doi.org/10.1016/j.cellbi.2009.01.019)

Twumasi P, Iakimova ET, Qian D. et al (2010a) Delayed programmed cell death affects the kinetics and dimensions of tracheary elements in xylogenic zinnia *(Zinnia elegans)* cells. BMC Plant Biol 10:162. doi: 10.1186/1471-2229-10-162

Twumasi P, Schel J, van Ieperen W (2010b) Osmotic potential of *Zinnia elegans* plant material affects the yield and morphology of tracheary elements produced *in vitro*. African J Biotechnol 9:8712-8721. doi: 10.5897/ajb10.1150

Weir IE, Maddumage R, Allan AC, Ferguson IB (2005) Flow cytometric analysis of tracheary element differentiation in *Zinnia elegans* cells. Cytometry Part A 68A:81-91. doi: 10.1002/cyto.a.20194

Yamamoto R, Fujioka S, Demura T, Takatsuto S, Yoshida S, Fukuda H (2001) Brassinosteroid levels increase drastically prior to morphogenesis of tracheary elements. Plant Physiol 125:556-563. doi: [​10.​1104/​pp.​125.​2.​556](http://dx.doi.org/10.1104/pp.125.2.556)

Ye Z-H, Varner JE (1996) Induction of cysteine and serine proteases during xylogenesis in *Zinnia elegans*. Plant Mol Biol 30:1233-1246. doi: 10.1007/bf00019555
