# Supplementary material for: Xylogenesis in zinnia (Zinnia elegans) cell cultures: unravelling the regulatory steps in a complex developmental programmed cell death event
Source: Planta. 2017 Feb 13;245(4):681–705. doi: 10.1007/s00425-017-2656-1 (PMC5357506; doi:10.1007/s00425-017-2656-1)
Supplement: Supplementary file 3 — Supplementary material 3 (DOCX 51 kb) [file 425_2017_2656_MOESM3_ESM.docx]

Title: **Xylogenesis in zinnia (*Zinnia elegans*) cell cultures: unravelling the regulatory steps in a complex developmental programmed cell death event**

Journal: Planta

Authors: Elena T. Iakimova^1^, Ernst J. Woltering^2,3^

^1^Institute of Ornamental Plants, Sofia, Bulgaria, ^2^Wageningen University and Research, Food & Biobased Research and ^3^Wageningen University, Horticulture and Product Physiology, Wageningen, The Netherlands

Corresponding author: Ernst J. Woltering

e-mail: ernst.woltering@wur.nl

**Microscopy for identification of the cellular structures and cell death signalling intermediates in xylogenic zinnia cell culture**

The morphological features of the living, dead, transdifferentiating and differentiated cells in zinnia culture can be reliably identified by means of various microscopy techniques. These include the use of various labelling probes for identification of e.g. the composition and structure of SCWs, microtubule organization, cytoplasm membrane system, and detection of the occurrence of cell death process including observations on vacuole expansion, changes in tonoplast permeability and vacuole rupture, nucleus and DNA disintegration, autolysis, phenotypic appearance of immature and mature TEs.

For example, staining with fluorescein diacetate (FDA) that is converted to a green fluorescing compound by the esterases in the living cells (Rotman and Papermaster 1966) is used for determining the viability of cultured mesophyll cells (Kuriyama 1999; Obara et al. 2001; Twumasi et al. 2009, 2010a). This fluorophore enters the cell in a passive way and becomes trapped inside the cell after its enzymatic breakdown to fluorescein in the cytoplasm (Rotman and Papermaster 1966). Typical for the living cells are FDA positive cytoplasm, diffuse nucleus and FDA negative intact vacuole. Kuriyama (1999) successfully applied this dye for observations on tonoplast permeability of differentiating TEs. With advancement of TE differentiation FDA was progressively taken up into the vacuole thus making possible to visualise the loss of tonoplast integrity. The author categorized the FDA stained zinnia cells as: living mesophyll cells (green fluorescence in the cytoplasm and intact FDA negative vacuole), living TEs with intact vacuole membrane and initial deposited SCWs (green fluorescence in cytoplasm), dying TEs with vacuole content mixed with the cytoplasm (yellow fluorescence in the whole cell) and dead TEs being hollow cells (no fluorescence). Living TEs with FDA positive intact cytoplasm, partially completed SCWs and FDA negative intact vacuole have been described also in the studies of Twumasi et al. (2010a). In other investigations it has been shown that the vital zinnia cells and the intact nucleus can be visualized by green fluorescent SYTO16 which is a membrane permeable dye that binds to nucleic acids (Obara et al. 2001). The nuclei in immature TEs are well distinguishable by the red fluorescence emitted from propidium iodide (PI), a fluorochrome permeable through the damaged plasma membrane and nuclear envelope (Obara et al. 2001; Twumasi et al. 2010a). In earlier experiments Weir et al. (2005) applied PI to detect chromatin condensation and DNA disintegration in cell death stage of zinnia TE formation *in vitro*. Twumasi et al. (2010a) used double FDA-PI staining for examination of immature TEs that showed PI positive compact nucleus, incomplete SCWs and fading FDA fluorescence remaining from partially digested cytoplasm mixed with the content of vacuole released after tonoplast rupture. Propidium iodide was shown also suitable for labelling of cell walls including SCWs in zinnia TEs (Pighin et al. 2004; Cattolin et al. 2009; Twumasi et al. 2010a). DNA double strand breaks that occur in the TE cell death stage can be detected by TUNEL (Terminal deoxynucleotidyl transferase mediated dUTP Nick End Labeling). TUNEL is a marker of free 3’-OH ends of DNA that accumulate during different kinds of cell death processes. This technique may not well discriminate the apoptotic-like internucleosomal DNA fragmentation and the random DNA cleavage (Gavrieli et al. 1992) but when considered in combination with other PCD markers (e.g. DNA laddering and cell morphological features) it can be reliably informative for identification of the cells in which PCD has actually taken place. Twumasi et al. (2010a) showed that TE maturation in zinnia cell culture was associated with DNA fragmentation evidenced by appearance of TUNEL positive nuclei. For example, TUNEL assay also revealed that DNA fragmentation occurs before the synthesis of the secondary cell wall in in *planta* differentiating xylem cells in *Populus* roots (Bagniewska-Zadworna et al. 2014). Various other kinds of DNA staining are known, but are rarely reported for determination of DNA degradation occurring during TE cell death in zinnia cell culture.

A classical dye to distinguish SCWs is Calcofluor White (CFW), which binds to the cellulose bands of the SCWs which following the labelling appear in blue colour. For observing the SCWs of zinnia TEs, CFW was for the first time introduced by Falconer and Seagull (1985, 1988). Since then this probe is widely applied to distinguish the cell wall thickenings in transdifferentiating zinnia cells (Groover and Jones 1999; Weir et al, 2005; Twumasi et al. 2010a; Novo-Uzal et al. 2013; Pesquet et al. 2013, and others). Secondary walls are also well distinguishable through staining of lignin with phloroglucinol (a dye that reacts with coniferyl aldehyde and cinnamaldehyde groups in lignin to yield red coloured cationic chromatophore) and by lignin and monolignols autofluorescence (Taylor et al. 1992; Groover and Jones 1999; Weir et al. 2005; Lacayo et al. 2010; Donaldson and Radotic 2013; Novo-Uzal et al. 2013; Pesquet et al. 2013; Tobimatsu et al. 2013). SCW thickenings can be visualized also by epifluorecence microscopy following staining with Tinopal LPW fluorescent brightener (a stilbene - an isomer of 1,2-diphenylethen) (Roberts and Haigler 1994).

Based on light microscopy of CFW stained zinnia cultured cells, [Kákošová](http://link.springer.com/search?facet-author=%22Anna+K%C3%A1ko%C5%A1ov%C3%A1%22) et al*.* (2013) classified differentiated TEs by their thickening. Vessel elements with annular, annular-helical, and helical SCW patterning were determined as protoxylem (PX)-like, those with helical-reticulate and reticulate as reticulate (R)-like and scalariform, reticulate-pitted and pitted as MX-like. The cell cultures were isolated from palisade and spongy mesophyll and the patterning was preserved in the cells that differentiated either without cell division or with division in longitudinal and transversal direction. The percent of PX-like and MX-like TEs originating from palisade parenchyma was comparable whereas from the TEs differentiated from spongy mesophyll cells 98 % were MX-like and the rest developed PX-like thickening.

The morphology of xylem elements, SCW architecture and cell death features have been accurately identified by TEM and atomic force microscopy (AFM). For example, AFM in combination with synchrotron radiation-based (SR)-FTIR spectromicroscopy was used for examination of the chemical composition and the arrangement of cellulose microfibrils of SCWs in zinnia TEs, labelled with carbohydrate-binding module which is a polysaccharide-specific probe (Lacayo et al. 2010). In TEM micrographs of sections of *Populus* stems Courtois-Moreau et al. (2009) have distinguished TE maturation associated autophagic-like features such as formation of small vesicles and dilatation of the ER after tonoplast rupture. The electron microscopy also revealed details of micro-autolysis within the intact central vacuole and of mega-autolysis. The latter is initiated following the disruption of tonoplast in differentiating vessel elements in *Arabidopsis* roots (Avci et al. 2008) and, various other cellular changes associated with SCW deposition and cell death execution in stem vascular tissue of the same plant (Turner et al. 2007). Early information collected through TEM analysis of differentiating zinnia TEs comes from the work of Burgess and Linstead (1984). Based on ultrastructural cellular features, they determined three phases of xylem element development in zinnia *in vitro* and in young leaves: cell wall thickening, cytoplasm degeneration and cell wall hydrolysis. Although in general the process is considered very similar *in vitro* and *in vivo*, these authors observed differences in ER system during SCW formation. In intact leaves swollen ER cisternae were positioned closely to the base of wall thickening but this was not observed in *in vitro* differentiating zinnia TEs*.* However, a similar pattern of microtubule redistribution before appearance of wall thickening and ER swelling at the inception of cytoplasm degeneration was seen in both conditions. Peculiarities of the hydrolysis of primary cell wall at the end of TE differentiation *in vitro* and *in planta* have been established. TEM images have shown that *in vivo* the hydrolysis leads to formation of loosely fibrillar wall which swells into the lumen of the cell where it disintegrates, whereas the hydrolysed wall of *in vitro* differentiating cells appeared granular lacking fibrillar components (Burgess and Linstead 1984). Haigler and Brown (1986) reported an electron microscopy study pointing to a critical role of Golgi apparatus in the synthesis and deposition of SCW components and export of cellulose synthesizing apparatus in zinnia cells transdifferentiating in culture. They detected rosette-shaped structures comprising particles thought to be important in cellulose synthesis. The rosettes were first localised in protoplast and with advancement of SCW formation found in plasma membrane over regions of secondary wall thickening but not between the bands. This suggested that rosettes might be continuously delivered and inserted into the membrane until completion of cell wall thickening. Examination by TEM of zinnia TEs in zinnia culture treated with cellulose synthesis inhibitors 2,6,-dichlorobenzonitrile (DCB) and isoxaben (herbicide) identified TEs with less regular SCW patterning. Interestingly, these TEs underwent normal cell death and autolysis. The disturbed thickening was associated with a lack of the hemicellulose xylan, the latter evidenced by absence of labelling with specific xylanase or antibody to xylan (Taylor et al. 1992). Xylan is suggested to participate in the linking of lignin to cellulose. In the same studies, differential interference contrast microscopy (phologlucinol/HCL staining of lignin and Tinopal LPW staining of cellulose in SCW) showed that despite DCB-induced changes in SCW thickening, lignin was in dispersed phase, in contrast to control TEs in which lignin was specifically localized in the SCW thickenings. These data were among the first suggesting a mechanism of SCW assembly which is dependent on supply of mediator molecules such as xylan and that the localization of cellulose might determine the localization of lignin (Taylor et al. 1992).

***Identification of cell death signalling intermediates***

The TE development in zinnia culture occurs in highly oxidative state (Barceló 1998a, b, 1999; 2005; Gómez-Ros et al. 2006; Novo-Uzal et al. 2013) the level of which is dependent on ROS production and detoxication by the cellular enzymatic and non-enzymatic antioxidant system. The involvement of ROS and especially H_2_O_2_ in PCD is well established (Levine et al. 1994). In the differentiation of xylem tissue, H_2_O_2_ is required for lignification (Novo-Uzal et al. 2013 and references therein). It is involved in peroxidase-mediated oxidative polymerization of cinnamyl alcohols to lignins and in the reinforcement of the cell wall through participating in cross-linking of cell wall proteins (Ogawa et al. 1997; Olson and Varner 1993; Levine et al. 1994; Barceló 1998a, b and references therein). In the work of Gómez-Ros et al. (2006), in zinnia culture and in vascular bundles in young zinnia stem H_2_O_2_ generation was visualized by CLSM using cyto- and histochemical staining with 2’-7’ dichlorofluorescein diacetate (DCFH-DA). Additionally, the presence of H_2_O_2_ was confirmed biochemically by xylenol orange, and superoxide anion (O_2_^-^) production was analysed with 2,3-bis(2-methoxy-4-nitro-5-sulfophenyl)-2H-tetrazolium-5-carboxanilide. In the same studies the crystalline thickening of SCWs of vascular cells in zinnia stem was imaged under polarized light microscopy and H_2_O_2_ distribution in cultured TEs was detected in electron micrographs. The observations have indicated that transdifferentiating living cultured cells are capable of producing ROS which occurs in nonlignifying (parenchyma-like) undifferentiated cells and at the beginning of TE lignification. Hydrogen peroxide synthesis in the vital cells was suggested necessary for lignification in the later stage of SCW formation in the dead TEs (Gómez-Ros et al. 2006). In other experiments, detection of H_2_O_2_ in zinnia stem vascular vessels by staining with starch/KI reagent pointed to a similar role of H_2_O_2_ in xylem lignification (Olson and Varner 1993; Ferrer and Barceló 1999; Barceló 1998a, b, 2005). Contribution of oxidative factors to TE differentiation process in zinnia cell culture was shown also earlier by Weir et al. (2005). They combined fluorescent microscopy with flow cytometry to analyse H_2_O_2_ production by staining with DCFH-DA and used monochlorobimane fluorescence for establishing the content of reduced glutathione.

By electron microscopy in CeCl_3_ stained zinnia mesophyll cells electron-dense deposits indicative for H_2_O_2_ production and localization have been viewed. In the early transdifferentiation stage the electron-dense granules were located on cell surfaces including cell wall and plasma membrane and in the later stage appeared uniformly distributed in the primary and secondary cell walls (Barceló 2005; Gómez-Ros et al. 2006). The findings provided additional evidence for participation of H_2_O_2_ in SCW lignification of zinnia xylem elements and suggested that H_2_O_2_ might be supplied from intercellular spaces.

A possible role of the gaseous hormone NO in xylem differentiation has been shown. CLSM of zinnia stem sections incubated with the NO-sensitive ﬂuorescent dye 4,5-diaminofluorescein-2 diacetate revealed a gradient of NO production which was low in mesophyll cells at the stage of acquisition of competence to transdifferentiate, highest during the process of transdifferentiation in accordance with SCW synthesis and during autolysis, and lowest in fully differentiated and ligniﬁed TEs (Gabaldon et al. 2005; Novo-Uzal et al. 2013). These findings demonstrated the contribution of NO to lignification and cell death.

**References**

Avci U, Petzold HE, Ismail IO, Beers EP, Haigler CH (2008) Cysteine proteases XCP1 and XCP2 aid micro-autolysis within the intact central vacuole during xylogenesis in *Arabidopsis* roots. Plant J 56:303-315. doi: 10.1111/j.1365-313x.2008.03592.x

Bagniewska-Zadworna A, Arasimowicz-Jelonek M, Smoliński DJ, Stelmasik A (2014) New insights into pioneer root xylem development: evidence obtained from *Populus trichocarpa* plants grown under field conditions. Ann Bot 113:1235-1247. doi: 10.1093/aob/mcu063

[Barceló](http://link.springer.com/search?facet-author=%22A.+Ros+Barcel%C3%B3%22) AR (1998a) The generation of H_2_O_2_ in the xylem of Zinnia elegans is mediated by an NADPH-oxidase-like enzyme. Planta 207:207-216. doi: 10.1007/s004250050474

Barceló AR (1998b) Hydrogen peroxide production is a general property of the lignifying xylem from vascular plants. Ann Bot 82:97-103. doi: 10.1006/anbo.1998.0655

[Barceló AR](http://europepmc.org/search;jsessionid=tntJjuBJLvWYTcO3SFMd.6?page=1&query=AUTH:%22Barcel%C3%B3+AR%22) (1999) Some properties of the H_2_O_2_/O_2_^-^ generating system from the lignifying xylem of *Zinnia elegans*. Free Radical Res 31 Suppl:S147-154. doi: 10.1080/10715769900301441

Barceló AR (2005) Xylem parenchyma cells deliver the H_2_O_2_ necessary for lignification in differentiating xylem vessels. Planta 220:747-756. doi: 10.1007/s00425-004-1394-3

Burgess J, Linstead P (1984) Comparison of tracheary element differentiation in intact leaves and isolated mesophyll cells of *Zinnia* *elegans*. Micron and Microsc Acta 15:153-160. [doi: 10.1016/0739-6260(84)90046-7](http://dx.doi.org.ezproxy.library.wur.nl/10.1016/0739-6260(84)90046-7)

Cattolin S, **Sorieul M, Hunter PR,**  **Khonsari RH, Frigerio L** (2009) *In vivo* imaging of the tonoplast intrinsic protein family in *Arabidopsis* roots. BMC Plant Biol 9: 133 doi: 10.1186/1471-2229-9-133

Courtois-Moreau CL, Pesquet E, Sjödin A et al (2009) A unique program for cell death in xylem fibers of *Populus* stem. Plant J 58:260-274. doi: 10.1111/j.1365-313x.2008.03777.x

Donaldson LA, Radotic K (2013) Fluorescence lifetime imaging of lignin autofluorescence in normal and compression wood. J Microsc 251:178-187. doi: 10.1111/jmi.12059

Falconer MM, Seagull RW (1985) Immunofluorescent and Calcofluor White staining of developing tracheary elements in *Zinnia* *elegans* L. suspension cultures. Protoplasma 125:190-198. doi: 10.1007/bf01281237

# Falconer MM, Seagull RW (1988) Xylogenesis in tissue culture III: Continuing wall deposition during tracheary element development. Protoplasma 144:10-16. doi: 10.1007/BF01320275

Ferrer MA, Barceló AR (1999) Differential effects of nitric oxide on peroxidase and H_2_O_2_ production by the xylem of *Zinnia elegans*. Plant Cell Environ 22:891-897. doi: 10.1046/j.1365-3040.1999.00459.x

Gabaldón C, Gómez-Ros LV, Pedreño MA, Barceló AR (2005) Nitric oxide production by the differentiating xylem of *Zinnia elegans*. New Phytolo 165:121-130. doi: 10.1111/j.1469-8137.2004.01230.x

Gavrieli Y, Sherman Y, Ben-Sasson SA (1992) Identification of programmed cell death *in situ* via specific labelling of nuclear DNA fragmentation. J Cell Biol 119:493-501.  doi: 10.1083/jcb.119.3.493

Gómez Ros LV, Paradiso A, Gabaldón C, Pedreño MA, de Gara L, Barceló AR (2006) Two distinct cell sources of H_2_O_2_ in the lignifying *Zinnia elegans* cell culture system. Protoplasma 227:175-183. doi: 10.1007/s00709-005-0128-0

Groover A, Jones AM (1999) Tracheary element differentiation uses a novel mechanism coordinating programmed cell death and secondary cell wall synthesis. Plant Physiol 119:375-384. doi: [​10.​1104/​pp.​119.​2.​375](http://dx.doi.org/10.1104/pp.119.2.375)

Haigler CH, Brown RM Jr (1986) Transport of rosettes from the Golgi apparatus to the plasma membrane in isolated mesophyll cells of *Zinnia elegans* during differentiation to tracheary elements in suspension culture. Protoplasma 134:111-120. doi: 10.1007/BF01275709

[Kákošová](http://link.springer.com/search?facet-author=%22Anna+K%C3%A1ko%C5%A1ov%C3%A1%22) A, Digonnet C, Goffner D. [Lišková](http://link.springer.com/search?facet-author=%22Desana+Li%C5%A1kov%C3%A1%22) D (2013) Galactoglucomannan oligosaccharides are assumed to affect tracheary element formation via interaction with auxin in *Zinnia* xylogenic cell culture. Plant Cell Rep 32:479-487. doi: 10.1007/s00299-012-1379-9

Kuriyama H (1999) Loss of tonoplast integrity programmed in tracheary element differentiation. Plant Physiol 121:763-774. doi: [​10.​1104/​pp.​121.​3.​763](http://dx.doi.org/10.1104/pp.121.3.763)

Lacayo CI, Malkin AJ, Holman HYN, et al (2010) Imaging cell wall architecture in single *Zinnia elegans* tracheary elements. Plant Physiol 154:121-133. doi: [10.1104/pp.110.155242](http://dx.doi.org/10.1104%2Fpp.110.155242)

Lee S, Woffenden BJ, Beers EP, Roberts AW (2000) Expansion of cultured mesophyll cells in response to hormones and light. Physiol Plantarum 108:216-222. doi: 10.1111/j.1399-3054.2011.01538.x

Levine A, Tenhaken R, Dixon R, Lamb C (1994) H_2_O_2_ from the oxidative burst orchestrates the plant hypersensitive disease resistance response. Cell 79:583-593. [doi:10.1016/0092-8674(94)90544-4](http://dx.doi.org/10.1016/0092-8674(94)90544-4)

Obara K, Kuriyama H, Fukuda H (2001) Direct evidence of active and rapid nuclear degradation triggered by vacuole rupture during programmed cell death in *Zinnia*. Plant Physiol 125:615-626. doi: [​10.​1104/​pp.​125.​2.​615](http://dx.doi.org/10.1104/pp.125.2.615)

Ogawa K, Kanematsu S, Asada K (1997) Generation of superoxide anion and localization of CuZn-superoxide dismutase in the vascular tissue of spinach hypocotyls: their association with lignification. Plant Cell Physiol 38:1118–1126.

Olson PD, Varner JE (1993) Hydrogen peroxide and lignfication. Plant J 4:887-892. doi: 10.1046/j.1365-313x.1993.04050887.x

Pesquet E, Zhang B, Gorzsas A, et al (2013) Non-cell-autonomous postmortem lignification of tracheary elements in Zinnia elegans. Plant Cell 25:1314-1328. doi: [10.1105/tpc.113.110593](http://dx.doi.org/10.1105%2Ftpc.113.110593)

Petzold HE, Zhao M, Beers EP (2012) Expression and functions of proteases in vascular tissues. Physiol Plantarum 145:121-129. doi: 10.1111/j.1399-3054.2011.01538.x

[Pighin](http://www.sciencemag.org/search?author1=Jamie+A.+Pighin&sortspec=date&submit=Submit) JA, [Zheng](http://www.sciencemag.org/search?author1=Huanquan+Zheng&sortspec=date&submit=Submit) H, [Balakshin](http://www.sciencemag.org/search?author1=Laura+J.+Balakshin&sortspec=date&submit=Submit) LJ, et al (2004) Plant cuticular lipid export requires an ABC transporter. Science 306:702-704. doi: 10.1126/science.1102331

Roberts AW, Haigher CH (1994) Cell expansion and tracheary element differentiation are regulated by extracellular pH

Rotman BB, Papermaster BW (1966) Membrane properties of living mammalian cells as studied by hydrolysis of fluorogenic esters. Proc Nat Acad Sci USA 55:134–141

Taylor JG, Owen TP Jr, Koonce LT, Haigler CH (1992) Dispersed lignin in tracheary elements treated with cellulose synthesis inhibitors provides evidence that molecules of the secondary cell wall mediate wall patterning. Plant J 2:959-970. doi: 10.1111/j.1365-313x.1992.00959.x

Tobimatsu Y, Wagner A, Donaldson L, et al (2013) Visualization of plant cell wall lignifications using fluorescence-tagged monolignols. Plant J 76:357-366. doi: 10.1111/tpj.12299

Turner S, Gallois P, Brown D (2007) Tracheary element differentiation. Annu Rev Plant Biol 58:407-433. doi: 10.1146/annurev.arplant.57.032905.105236

Twumasi P, Iakimova ET, Qian D. et al (2010a) Delayed programmed cell death affects the kinetics and dimensions of tracheary elements in xylogenic zinnia *(Zinnia elegans)* cells. BMC Plant Biol 10:162. doi: 10.1186/1471-2229-10-162

Twumasi P, Schel J, van Ieperen W (2010b) Osmotic potential of *Zinnia elegans* plant material affects the yield and morphology of tracheary elements produced *in vitro*. African J Biotechnol 9:8712-8721. doi: 10.5897/ajb10.1150

Weir IE, Maddumage R, Allan AC, Ferguson IB (2005) Flow cytometric analysis of tracheary element differentiation in *Zinnia elegans* cells. Cytometry Part A 68A:81-91. doi: 10.1002/cyto.a.20194
