# Supplementary material for: Xylogenesis in zinnia (Zinnia elegans) cell cultures: unravelling the regulatory steps in a complex developmental programmed cell death event
Source: Planta. 2017 Feb 13;245(4):681–705. doi: 10.1007/s00425-017-2656-1 (PMC5357506; doi:10.1007/s00425-017-2656-1)
Supplement: Supplementary file 4 — Supplementary material 4 (DOCX 54 kb) [file 425_2017_2656_MOESM4_ESM.docx]

Title: **Xylogenesis in zinnia (*Zinnia elegans*) cell cultures: unravelling the regulatory steps in a complex developmental programmed cell death event**

Journal: Planta

Authors: Elena T. Iakimova^1^, Ernst J. Woltering^2,3^

^1^Institute of Ornamental Plants, Sofia, Bulgaria, ^2^Wageningen University and Research, Food & Biobased Research and ^3^Wageningen University, Horticulture and Product Physiology, Wageningen, The Netherlands

Corresponding author: Ernst J. Woltering

e-mail: ernst.woltering@wur.nl

**Pharmacological studies contributing to elucidation of differentiation and cell death associated signalling in xylogenic zinnia cell culture**

In addition to the information provided in the main text, here the insights in the regulation of xylogenesis in cultured zinnia cells gained through pharmacological analyses are described.

***Auxin and cytokinin***

The formation of continuous vessel fibers *in planta* is dependent on polar auxin flow which in case of wounding is interrupted leading to disturbed mode of xylem development (Kuriyama and Fukuda 2000; Fukuda 2004; Mattsson et al. 1999 and references therein). The importance of auxin transport for canalization of xylem differentiation in a narrow column of cells (Sachs 2000) has been examined by application of the polar auxin transport inhibitors 1-N-naphthylphthalamic acid (NPA), 9-hydroxyfluorene-9-carboxylic acid (HFCA) and 2,3,5-triiodobenzonic acid (TIBA). In the presence of these chemicals the normal distribution and patterning of vascular bundles in *Arabidopsis* leaf parenchyma cells have been affected (Mattsson et al. 1999). In intact leaves exposed to wounding or in conditions of vascular blockage which interrupts the water flow, the continuity of xylem tissue is restored by development of xylem conduits bypassing the injured areas. The TEs composing the new routes arise from parenchyma cells surrounding the wound (Aloni 1987; Fukuda 1992). In *Arabidopsis*, *Pinus pinea* and other species the ability of leaf parenchyma cells to transdifferentiate and form rescue xylem conduits has been attributed to a leakage of auxin from wounded cells (Aloni 1987; Kalev and Aloni 1998a, b; Mattsson et al. 1999). Similarly, in xylogenic zinnia culture, auxin released from wounded mesophyll cells is suggested to stimulate the transdifferentiation (Fukuda 1992; Kuriyama and Fukuda 2000). This has been corroborated through experiments with the auxin transport inhibitors NPA, HFCA and TIBA. Treatments of zinnia cell culture with the inhibitors prevented auxin efflux from the cells and suppressed the transdifferentiation. The process was associated with an increase of total NAA amount but with a depletion of intracellular free NAA due to enhanced NAA metabolism. In the presence of NPA the transition from stage I to stage II was inhibited thus suggesting a contribution of auxin transport to early transdifferentiation events (Yoshida et al. 2005). In zinnia xylogenic culture, TE differentiation was also prevented in medium supplemented with anti-auxins *p*-chlorophenoxyisobutyric acid or *p*-chlorophenoxyisobutyric acid (Church and Galston 1988).

Little information is available about inhibitory studies related to cytokinins. Some early experiments of Church and Galston (1988) have shown that the addition of anti-cytokinins (4-(cyclopentylamino)-2- methylthiopyrrolo[2,3-*d*]pyrimidine) and (3-methyl-7-*n*-pentylaminopyrazolo [4,3-*b*]pyridine) suppressed TE development if added at day 3 of culture initiation but did not affect the division of mesophyll cells. It has not been tested whether the inhibition of TE differentiation caused by anticytokinins can be reversed by increasing the cytokinin concentration in the culture medium.

***Gibberellin***

Few pharmacological analyses have addressed the role of gibberellins in zinnia xylogenesis. A contribution of GA3 to lignification of differentiating zinnia TEs has been shown in experiments with inhibitors of GA3 metabolism. It was found that lignification is suppressed in response to treatment of the culture with ancymidol, paclobutrazol and uniconazole that are known to inhibit the first steps of GA3 synthesis pathway, namely the conversions from ent-kaurene to ent-kaurenoic acid which are catalyzed by cytochrome P450-dependent monooxygenase (Tokunaga et al. 2006). The effect of inhibitors has been reversed at the addition of GA3 and BRs. As paclobutrazol and uniconazole are also inhibitors of BRs and sterol synthesis these studies provided information also about BRs involvement in xylogenesis.

***Ethylene***

The gaseous plant hormone ethylene is involved in the mediation of zinnia TE differentiation*.* Exposure of zinnia culture to ethylene precursor 1-aminocyclopropane-1-carboxylic acid (ACC) and ethylene-releasing compound 2-chloroethyl-phosphonic acid (ethephon) potentiated the TE formation whereas the addition of inhibitors of ethylene synthesis such as CoCl_2_ and 1-aminoisobutyric acid (AIB) (both inhibitors of ACC oxidase (ACO)), and aminoethoxyvinylglycine (AVG), an inhibitor of ACC synthase (ACS) suppressed the process. This suggested that ethylene production is a prerequisite for zinnia TE development (Pesquet and Tuominen 2011). The application of ethylene receptor blocker silver thiosulfate (STS) resulted in inhibition of both TE cell death and TE lignification thus showing a role of ethylene perception in the transdifferentiation process in this cell system (Pesquet et al. 2013).

***Nitric oxide***

Pharmacological analyses have also shown the involvement of NO in transdifferentiation. Exposure of zinnia cells to NO scavenger 2 -phenyl-4,4,5,5-tetramethyl imidazoline-1-oxyl-3-oxide (CPTIO) led to strong suppression of transdifferentiation and cell death which indicated the role of NO in the processes of SCWs synthesis and PCD (Gabaldón et al. 2005). Ferrer and Barceló (1999) performed well designed experiments to study the interaction of NO with peroxidase in lignifying xylem fibres in zinnia stems. These authors treated the stems with the NO releasing compounds sodium nitroprusside (SNP) or *S*-nitroso-*N*-acetyl-penicillamine or with NO gas, detected H_2_O_2_ with starch/KJ reagent, histochemically determined peroxidise activity by using the chromogenic substrate 3,3',5,5'-tetramethylbenzidine and by isoelectric focusing and, distinguished peroxidise isoenzymes by staining with 4-methoxy-a-naphthol. It was found that NO-releasing compounds and NO itself suppressed the activity of coniferyl alcohol peroxidase which is a basic peroxidise present in intercellular washing fluid of zinnia. Treatments of the cell culture with 5.0 mmol m^-3^ SNP (releasing approximately 35 µmol NO m^-3^ s^-1^) and with NO gas in concentrations of 55 or 100 µmol m^-3^ s^-1^ have inhibited the enzyme activity by 42 %, 62 % and 100 %, respectively. However, the chemicals did not affect H_2_O_2_ production in lignifying xylem *in planta*. These results suggested that NO may contribute to lignification through directly or indirectly interfering with non-enzymatic factors or with other enzymes (such as NADPH oxidase and superoxide dismutase) responsible for synthesis and conversion of other ROS preceding the H_2_O_2_ generation.

***Calcium and other signals***

# Zinnia TE differentiation is dependent on Ca^2+^ and CaM signalling. This has been demonstrated following application of the Ca^2+^ channel blockers La^3+^ and dihydropyridine, and the CaM inhibitors trifluoperazine (TFP), chlorpromazine (CP), tetracaine and N-(6-aminohexyl)-5-chloro-1-naphthalenesulfonamide hydrochloride (W-7). The agents suppressed TE formation if administrated together with auxin and CK. These experiments suggested that Ca^2+^/CaM system is involved in a relatively early stage of transdifferentiation. Calmodulin binds to CaM binding proteins in the presence of Ca^2+^. An increase of the levels of at least two of CaM binding proteins of 27 and 28 kDa as well as the levels of CaM and membrane bound Ca^2+^ has been established prior to the onset of SCWs deposition (Roberts and Haigler 1990; Kobayashi and Fukuda 1994). Additionally, the application of CaM antagonists at the beginning of SCWs deposition inhibited the aggregation of actin filaments in the cytoskeleton which is related to reorganisation of microtubules predefining the architecture of SCW thickening (Kobayashi and Fukuda 1994 and references therein). Complete inhibition of TE differentiation in zinnia cell culture has been reported also at treatment with calcium-channel blocker nifedipine if added to the culture 2-3 h before the beginning of SCW deposition (Roberts and Haigher 1992). Contrary, in the same study it was found that the methylxanthines caffeine and theophylline that stimulate the release of Ca^2+^ from intracellular stores are able to inhibit TE differentiation but only if applied at least 8 h prior to the appearance of cell wall thickenings. These results suggested that soon before the onset of SCW deposition TE differentiation is dependent on the influx of extracellular calcium whereas earlier events might be associated with intracellular Ca^2+^ efflux.

Inhibitory studies have revealed more regulatory elements of TE differentiation in zinnia cell culture. For example, application of α-aminooxy-β-phenylpropionic acid, an inhibitor of PAL activity, blocked lignin synthesis in zinnia cells suggesting a contribution of phenylpropanoid pathway to lignin production (Ingold et al. 1990)*.* Suzuki et al. (1992) demonstrated that inhibition of cellulose synthesis with 2,6-dichlorobenzonitrile disturbed the positioning of lignin deposits and the thickenings of *in vitro* developing zinnia TEs. Through administration of a range of compounds such as protein phosphatase inhibitor okadaic acid, protein kinase inhibitor staurosporine, protein synthesis inhibitor cycloheximide, RNA synthesis inhibitor actinomycin D, heterotrimeric G-protein activator mastoparan, Ca^2+^ ionophore A23187, respiration inhibitor sodium azide and others, the contribution of various signalling pathways to formation of zinnia vessel elements *in vitro* has been recognized (Groover et al. 1997; Groover and Jones 1999). Barceló (1999) applied the calmodulin (CaM) inhibitor 1-[bis(p-chlorophenyl)methyl]-3-[2,4-dichloro-β-(2,4-dichlorobenzyloxy)phenethyl]imidazoliniumchloride), phospholipase C inhibitor [neomycin sulfate](http://europepmc.org/abstract/med/10694053/?whatizit_url_Chemicals=http://www.ebi.ac.uk/chebi/searchId.do?chebiId=CHEBI%3A31635) and  [staurosporine](http://europepmc.org/abstract/med/10694053/?whatizit_url_Chemicals=http://www.ebi.ac.uk/chebi/searchId.do?chebiId=CHEBI%3A15738). These substances compromised the lignification and diminished the levels of produced ROS which was restored at the addition of the protein phosphatase inhibitor cantharidin. The obtained results indicated that the activation of the enzymatic system responsible for generation of [H_2_O_2_](http://europepmc.org/abstract/med/10694053/?whatizit_url_Chemicals=http://www.ebi.ac.uk/chebi/searchId.do?chebiId=CHEBI%3A30492)/[O_2_^-^](http://europepmc.org/abstract/med/10694053/?whatizit_url_Chemicals=http://www.ebi.ac.uk/chebi/searchId.do?chebiId=CHEBI%3A18421,29356) in lignifying xylem of [zinnia](http://europepmc.org/abstract/med/10694053/?whatizit_url_Species=http://www.ncbi.nlm.nih.gov/Taxonomy/Browser/wwwtax.cgi?id=34245&lvl=0) plant is exerted through a complicated network in which CaM, phospholipid signalling, and protein phosphoryllation are involved.

***DNA synthesis***

Pharmacological analyses have indicated that DNA synthesis is not strictly necessary for the cells to enter the differentiation process. The application of DNA synthesis inhibitors 5-fluorodeoxyuridine and colchicine has not affected the transdifferentiation of non-divided cells but suppressed the differentiation of TEs originating from dividing cells (Dodds 1980). Further, in other studies has been found that other agents known to inhibit DNA synthesis such as aphidicolin, arabinosyl cytosine, fluorodeoxyuridine, fluorouracil and mitomycin C prevented the transdifferentiation (Sugiyama and Komamine 1987). [Kákošová](http://link.springer.com/search?facet-author=%22Anna+K%C3%A1ko%C5%A1ov%C3%A1%22) et al. (2013) showed that following cell division the mesophyll cells are capable to transdifferentiate into TEs. Earlier, Iwasaki et al. (1986) reported that GA3 may inhibit the cell division and DNA synthesis in isolated zinnia mesophyll cells but whether this is related to transdifferentiation was not discussed.

***Proteolytic enzymes***

# Some of the first suggestions concerning the involvement of proteolytic activities in zinnia TE differentiation cascade *in vitro* are as well based on pharmacological studies. Application of a range of inhibitors provided evidence for participation of cysteine and serine proteases in the hydrolysis of the cellular content in differentiating TEs. For example, the application of the irreversible cysteine protease inhibitor L- transepoxysuccinyl-leucylamido- [4-guanidino]butane (E64) and serine protease inhibitor phenylmethylsulfonyl fluoride suppressed the TE formation in zinnia cell culture. The proteins were identified as 29 kD cysteine and 60 kDa serine proteases. A cysteine protease activity was induced *in vitro* and corresponded to cysteine protease activity present in the xylem of zinnia stems (Ye and Varner 1996). Additionally, the participation of cysteine proteases in transdifferentiation of cultured zinnia cells was substantiated through the inhibition of TE production *in vitro* by E64 (Twumasi et al. 2010a). The existence of cysteine endopeptidase with molecular mass of 30 kDa contributing to transdifferentiation of zinnia mesophyll cells into immature TEs has been suggested through repression of its activity by the irreversible cysteine protease inhibitor [L-3-*trans*-carboxyoxiran-2-carbonyl]-L-Leu-agmatin (Minami and Fukuda 1995). The involvement of a wide range of cysteine proteases was indicated also by the complete inhibition of transdifferentiation occurring in presence of the broad range cysteine protease inhibitors N-ethylmaleimide and iodoacetamide (Iakimova and Woltering 2009). In their experiments Groover and Jones (1999) treated the zinnia culture with trypsin which triggered TE cell death. Following application of trypsin inhibitors, SCW formation, TE cell death and autolysis were suppressed. In the same study it was also shown that, after finalization of SCW deposition, a cell wall localized cell death inducing serine protease stimulated Ca^2+^ influx. These results showed that SCW and PCD are interconnected. Woffenden et al. (1998) have applied the proteasome inhibitors *clasto*-lactacystin β-lactone (LAC) and carbobenzoxy-leucinyl-leucinyl-leucinal (LLL) at the time of zinnia cell culture initiation. The former irreversibly inhibits tryptic, chymotryptic, and peptidylglutamic cleavage activities of the proteasome. In addition to being a proteasome inhibitor, LLL is also a calpain Ca^2+^-dependent thiol protease inhibitor. When these two agents were added before induction of the culture they totally prevented the transdifferentiation whereas if applied post-induction with auxin and CK, only LAC delayed the TE development. An increase of the portion of living TEs and dead TEs expressing incomplete autolytic clearance of cellular content was observed at the application of LLL. Further evidence for proteasome involvement in xylem cell death came from Han et al. (2012) who, by using LAC, managed to inhibit the differentiation of vessel elements in an *Arabidopsis* xylogenic culture. These results pointed to a regulatory role of the proteasome and of cysteine proteases in autolytic elimination of the cellular content in *in vitro* developing TEs.

# Plant caspase-like proteases (CLP) are thought to play an important role in most PCD processes in plants. These enzymes are functional but not structural homologues to the animal cysteinyl asparic proteases (caspases) acting as cell death executioners (Woltering 2010; Petzold et al. 2012 and references therein). Through employing pharmacolocological analysis Twumasi et al. (2010a) reported the first observation on possible CLPs contribution to zinnia transdifferentiation/PCD *in vitro*. These authors showed that the irreversible broad-ranged human caspase inhibitor benzyoxycarbonyl-Asp-2,6-dichlorobenzoyloxymethylketone (Z-Asp-CH2-DCB), the irreversible caspase-1 inhibitor Tyr-Val-Ala-Asp-chloromethylketone (Ac-YVAD-CMK), and the reversible caspase-3 tetrapeptide inhibitor Acyl-Asp-Glu-Val-l-aspartic acid aldehyde (Ac-DEVD-CHO) delayed TE cell death which corresponded to a delay in the timing of the formation of completed TEs. The inhibitors also suppressed the occurrence of the PCD markers DNA laddering and appearance of TUNEL positive nuclei.

The above discussion shows the efficiency of pharmacological approaches in the elucidation of the most important findings on the signalling processes mediating the xylogenesis in zinnia *in vitro*.

**References**

Aloni R (1987) Differentiation of vascular tissues. Annu Rev Plant Physiol 38:179-204. doi: 10.1146/annurev.pp.38.060187.001143

[Barceló AR](http://europepmc.org/search;jsessionid=tntJjuBJLvWYTcO3SFMd.6?page=1&query=AUTH:%22Barcel%C3%B3+AR%22) (1999) Some properties of the H_2_O_2_/O_2_^-^ generating system from the lignifying xylem of *Zinnia elegans*. Free Radical Res 31 Suppl:S147-154. doi: 10.1080/10715769900301441

Church DL, Galston AW (1988) Hormonal induction and antihormonal inhibition of tracheary element differentiation in Zinnia cell cultures. [Phytochemistry](http://www.sciencedirect.com/science/journal/00319422) 27:2435-2439.

# Dodds JH (1980) The effect of 5-fluorodeoxyuridine and colchicine on tracheary element differentiation in isolated mesophyll cells of *Zinnia elegans* L. Z Pflanzenphysiol 99:283-285. [doi:10.1016/S0044-328X(80)80141-3](http://dx.doi.org/10.1016/S0044-328X(80)80141-3)

Ferrer MA, Barceló AR (1999) Differential effects of nitric oxide on peroxidase and H_2_O_2_ production by the xylem of *Zinnia elegans*. Plant Cell Environ 22:891-897. doi: 10.1046/j.1365-3040.1999.00459.x

Fukuda H (1992) Tracheary element formation as a model system of cell differentiation. In: Jeon KW, Friedlander M (eds) Interantional Review of Cytology, vol 136, Academic Press Inc, San Diego, California, pp 289-332. ISBN: 0-12-364536-0

Fukuda H (2004) Signals that control plant vascular cell differentiation. Nature Rev Mol Cell Biol 5:379-391. doi:10.1038/nrm1364

Gabaldón C, Gómez-Ros LV, Pedreño MA, Barceló AR (2005) Nitric oxide production by the differentiating xylem of *Zinnia elegans*. New Phytolo 165:121-130. doi: 10.1111/j.1469-8137.2004.01230.x

# Greenberg JT (1996) Programmed cell death: A way of life for plants. Proc Natl Acad Sci USA 93:12094-12097.

Groover A, DeWitt N, Heidel A, Jones A (1997) Programmed cell death of plant tracheary elements differentiating *in vitro*. Protoplasma 196:197-211. doi: 10.1007/BF01279568

Groover A, Jones AM (1999) Tracheary element differentiation uses a novel mechanism coordinating programmed cell death and secondary cell wall synthesis. Plant Physiol 119:375-384. doi: [​10.​1104/​pp.​119.​2.​375](http://dx.doi.org/10.1104/pp.119.2.375)

Haigler CH, Brown RM Jr (1986) Transport of rosettes from the Golgi apparatus to the plasma membrane in isolated mesophyll cells of *Zinnia elegans* during differentiation to tracheary elements in suspension culture. Protoplasma 134:111-120. doi: 10.1007/BF01275709

Han J-J, Lin W, Oda Y, Cui K-M, Fukuda H, He X-Q (2012) The proteasome is responsible for caspase-3-like activity during xylem development. Plant J 72:129-141. doi: 10.1111/j.1365-313x.2012.05070.x

Iakimova ET, Woltering EJ (2009) Modulation of programmed cell death in a model system of xylogenic zinnia (*Zinnia elegans*) cell culture. Biotechnol Biotec Eq 23(SE):542-546. doi: 10.1080/13102818.2009.10818482

Ingold E, Sugiyama M, Komamine A (1990) L-a-aminooxy-b-phenylpropionic acid inhibits lignification but not the differentiation to tracheary elements of isolated mesophyll cells of *Zinnia elegans.* Physiol Plantarum 78:67-74. doi: 10.1111/j.1399-3054.1990.tb08716.x

Iwasaki T, Fukuda H, Shibaoka H (1986) Inhibition of cell division and DNA synthesis by gibberellin in isolated Zinnia mesophyll cells. Plant Cell Physiol 27:717-724.

[Kákošová](http://link.springer.com/search?facet-author=%22Anna+K%C3%A1ko%C5%A1ov%C3%A1%22) A, Digonnet C, Goffner D. [Lišková](http://link.springer.com/search?facet-author=%22Desana+Li%C5%A1kov%C3%A1%22) D (2013) Galactoglucomannan oligosaccharides are assumed to affect tracheary element formation via interaction with auxin in *Zinnia* xylogenic cell culture. Plant Cell Rep 32:479-487. doi: 10.1007/s00299-012-1379-9

Kalev N, Aloni R (1998a) Role of auxin and gibberellin in regenerative differentiation of tracheids in *Pinus pinea* seedlings. New Phytol 138:461-468. doi: 10.1046/j.1469-8137.1998.00119.x

Kalev N, Aloni R (1998b) Role of ethylene and auxin in regenerative differentiation and orientation of tracheids in *Pinus pinea* seedlings. New Phytol 142:307-313. doi: 10.1046/j.1469-8137.1999.00398.x

Kobayashi H, Fukuda H (1994) Involvement of calmodulin and calmodulin-binding proteins in the differentiation of tracheary elements in *Zinnia* cells*.* Planta 194:388-394. doi: 10.1007/bf00197540

Kuriyama H, Fukuda H (2000) Regulation of tracheary element differentiation. J Plant Growth Regul 20:35-51. doi: 10.1007/s003440010006

Mattsson J, Sung RZ, Thomas Berleth T (1999) Responses of plant vascular systems to auxin transport inhibition. Development 126:2979-2991.

Minami A, Fukuda H (1995) Transient and specific expression of a cysteine endopeptidase associated with autolysis during differentiation of *Zinnia* mesophyll cells into tracheary elements. Plant Cell Physiol 36:1599-1606.

Pesquet E, Tuominen H (2011) Ethylene stimulates tracheary element differentiation in Zinnia elegans cell cultures. New Phytol 190:138-149. doi: 10.1111/j.1469-8137.2010.03600.x

Pesquet E, Zhang B, Gorzsas A, et al (2013) Non-cell-autonomous postmortem lignification of tracheary elements in Zinnia elegans. Plant Cell 25:1314-1328. doi: [10.1105/tpc.113.110593](http://dx.doi.org/10.1105%2Ftpc.113.110593)

Petzold HE, Zhao M, Beers EP (2012) Expression and functions of proteases in vascular tissues. Physiol Plantarum 145:121-129. doi: 10.1111/j.1399-3054.2011.01538.x

Roberts AW, Haigher CH (1990) Tracheary element differentiation in suspension-cultured cells of *Zinnia* requires uptake of extracellular Ca^2+^. Planta 180:502-509. doi: 10.1007/BF02411447

# Roberts AW, Haigher CH (1992) Methylxanthines reversibly inhibit tracheary-element differentiation in suspension cultures of *Zinnia elegans* L. Planta 186:586-592. doi: 10.1007/bf00198040

Sachs T (2000) Integrating cellular and organismic aspects of vascular differentiation. Plant Cell Physiol 41:649–656. doi: 10.1093/pcp/41.6.649

Sugiyama M, Komamine A (1987) Relationship between DNA synthesis and cytodifferentiation to tracheary elements. Oxford Surveys Plant Mol Cell Biol 4:343-346.

Suzuki K, Ingold E, Sugiyama M, Fukuda H, Komamine A (1992) Effects of 2,6 dichlorobenzonitrile on differentiation to tracheary elements of isolated mesophyll cells of *Zinnia elegans* and formation of secondary cell walls. Physiol Plantarum 86:43-48. doi: 10.1111/j.1399-3054.1992.tb01309.x

Tokunaga N, Uchimura, N, Sato Y (2006) Involvement of gibberellin in tracheary element differentiation and lignification in *Zinnia elegans* xylogenic culture. Protoplasma 228:179-187. doi: 10.1007/s00709-006-0180-4

Twumasi P, Iakimova ET, Qian D. et al (2010a) Delayed programmed cell death affects the kinetics and dimensions of tracheary elements in xylogenic zinnia *(Zinnia elegans)* cells. BMC Plant Biol 10:162. doi: 10.1186/1471-2229-10-162

Woffenden BJ, Freeman TB, Beers EP (1998) Proteasome inhibitors prevent tracheary element differentiation in Zinnia mesophyll cell cultures. Plant Physiol 118:419-430. doi: [​10.​1104/​pp.​118.​2.​419](http://dx.doi.org/10.1104/pp.118.2.419)

Woltering EJ (2010) Death proteases: alive and kicking. Trends Plant Sci 15:185-188. [doi:10.1016/j.tplants.2010.02.001](http://dx.doi.org/10.1016/j.tplants.2010.02.001)

Ye Z-H, Varner JE (1996) Induction of cysteine and serine proteases during xylogenesis in *Zinnia elegans*. Plant Mol Biol 30:1233-1246. doi: 10.1007/bf00019555

# Yoshida S, Kuriyama H, Fukuda H (2005) Inhibition of transdifferentiation into tracheary elements by polar auxin transport inhibitors through intracellular auxin depletion. Plant Cell Physiol 46:2019-2028. doi: 10.1093/pcp/pci217
