# Supplementary material for: Xylogenesis in zinnia (Zinnia elegans) cell cultures: unravelling the regulatory steps in a complex developmental programmed cell death event
Source: Planta. 2017 Feb 13;245(4):681–705. doi: 10.1007/s00425-017-2656-1 (PMC5357506; doi:10.1007/s00425-017-2656-1)
Supplement: Supplementary file 5 — Supplementary material 5 (DOCX 47 kb) [file 425_2017_2656_MOESM5_ESM.docx]

Title: **Xylogenesis in zinnia (*Zinnia elegans*) cell cultures: unravelling the regulatory steps in a complex developmental programmed cell death event**

Journal: Planta

Authors: Elena T. Iakimova^1^, Ernst J. Woltering^2,3^

^1^Institute of Ornamental Plants, Sofia, Bulgaria, ^2^Wageningen University and Research, Food & Biobased Research and ^3^Wageningen University, Horticulture and Product Physiology, Wageningen, The Netherlands

Corresponding author: Ernst J. Woltering

e-mail: ernst.woltering@wur.nl

**Intercellular signalling**

In the xylogenic zinnia cell cultures, a portion of the cells do not differentiate. These are suggested to serve as feeder cells supplying substrates necessary for the transdifferentiation of the others. In turn, transdifferentiating cells (that are still alive) send messenger molecules to the non-differentiating cells to either initiate or prevent TE development.

Arabinogalactan (ARG)-like proteins, BRs, PSK, mono- and dilignols, some peptides and H_2_O_2_ have been identified as the main factors contributing to cell-to-cell signalling (Fig. 4, main text).

A nonclassical type of ARG protein is proposed to fulfil a function as intercellular communicator, first known as hypothetical ‘xylogen’. A medium from low-density cell culture has very low level or is lacking ARG but in medium from differentiating cells the content of this compound increases during the course of TEs formation. A positive feedback loop in which the immature xylem cells produce ARG which stimulates the pro-cambium cells to differentiate and the newly differentiating cells to produce more ARG which further stimulates the transdifferentiation of not yet differentiated cells was suggested (Motose et al. 2001a, b). In their later study Motose et al. (2004) identified the xylogen as a proteoglycan-like factor with hybrid-type molecule with properties of both ARG proteins and nonspecific lipid-transfer proteins. They named the gene *Zinnia elegans xylogen protein 1* (*ZeXYP1*). The transcripts were shown to accumulate 12 h before visible appearance of TEs and the levels reached a peak at 48 h after the induction of the culture. Arabinogalactan accumulation corresponded to stage II of xylogenesis in which the dedifferentiated cells become precursor TEs. It was also found that auxin stimulates the expression of this gene whereas both CK and auxin were required for accumulation of the protein (Motose et al. 2004). By using ZeXYP1 protein sequence as a query against the TAIR *Arabidopsis* database, Kobayashi et al. (2011) performed BLAST (basic local alignment search tool) search for xylogen-type genes in the *Arabidopsis* genome and identified 13 xylogen-type genes with significant similarity to *ZeXYP1,* responsible for encoding ARG-like proteins. Among them *AtXYP2*, appeared as the best candidate for a counterpart of *ZeXYP1*. Genes encoding for ARG-like proteins were as well identified in *Populus*, *Vitis*, *Lotus*, *Oryza*, *Selaginella* and *Physcomitrella*, suggesting that xylogen-type proteins might function as intercellular differentiation-related signals in various land plants (Kobayashi et al. 2011).

A role of ARG in the control over the arrangement of SCW thickenings in zinnia was proposed by Dahiya et al. (2006). From cDNA library, derived from induced to transdifferentiate cultured zinnia cells, these authors isolated a fasciclin-domain-containing *ZeFLA11* gene - a putative zinnia homologue of *Arabitdopsis* gene *FLA11* (fasciclin-like arabinogalactan protein; *At5g03170*) that was suggested to contribute to pattern formation of SCWs. *In situ* hybridization of *ZeFLA11* with an antisense ribioprobe with transverse sections, performed in isolated stem and leaf sections of zinnia seedlings, revealed that the gene expression corresponded with the formation of R-shaped thickening of differentiating MX vascular bundles and adjacent parenchyma cells in the stem and not with the development of SCW with spiral thickening pattern in the leaf vessels. These data indicated that this ARG (a glycosylphosphatidylinositol (GPI) anchored protein) may contribute to the formation of MX generated vessel elements with reticulate architecture of SCWs. In zinnia cell culture derived from palisade leaf mesophyll, Dahiya et al. (2005) isolated the gene ZeRH2.1 containing ring finger domain (really interesting new gene, RING) and encoding for RING-H2 protein. Expression of ZeRH2.1 was detected in freshly isolated mesophyll cells and in the time course of TE differentiation. Accumulation of abundant transcripts of ZeRH2.1 was observed in xylem parenchyma of leaf vascular bundles, in the companion cells of the stem fibers and in the phloem cells of juvenile vessels. The enhanced localization of ZeRH2.1 transcripts in this specific sites suggested that ZeRH2.1 may function as intercellular transporter involved in protein-protein interactions.

Brassinosteroids are suggested to serve as intercellular communicators operating in stages I and III of TE differentiation. In *Arabidopsis* it has been shown that the peptide is perceived at the cell surface by a membrane-bound receptor kinase of the leucine-rich repeat family. The receptor activity is regulated by calmodulin (Sauter 2015). In transdifferentiating zinnia cells it has been suggested that in early and late stages, different BRs may operate in extracellular signalling. For example, the level of castersterone in the culture medium increased significantly just before stage III suggesting its involvement in mediation of the entry into the cell death phase whereas brassinolide was stimulated by auxin and proposed to play a role in cell-to-cell interaction in the early transdifferentiation process (Motose et al. 2001a, b; Yamamoto et al. 2001, 2007). In similarity to ARG, PSK was present in conditioned medium collected from cultures with high cell density that promoted the transdifferentiation of cells at low density. And again, in a manner similar to ARG, a positive feedback loop in the regulation of initial transdifferentiation stage was suggested: PSK stimulates the transformation of dedifferentiated cells into pro-cambium-like cells which in turn produce more PSK, in this way maintaining the pro-cambium-like state (Motose et al. 2001a, b).

In zinnia and *Arabidopsis* xylogenic cultures and in zinnia stems the lignification of SCWs was determined as a partially non-autonomous process dependent on factors such as mono- and dilignols (acting as lignin precursors) and H_2_O_2_ being produced by the living non-transdifferentiating cells (Barceló 1998b; Groover et al. 1997; Hosokawa et al. 2001; Tokunaga et al. 2005; Avci et al. 2008; Bollhöner et al. 2012, 2013; Novo-Uzal et al. 2013; Pesquet et al. 2013; Schuetz et al. 2013; Farquharson 2014; Ménard and Pesquet 2015). Differential gene expression analysis of zinnia cells *in vitro* and *in vivo* identified genes encoding for the enzymes cinnamoyl CoA reductase and cinnamyl alcohol dehydrogenase that are involved in the synthesis of lignin monomers. The genes were expressed in both non-TE cells and in differentiating TEs. Lignin monomers are transported through the culture and utilized for TE lignification also in *post-mortem* stage (Pesquet et al*.* 2013; Ménard and Pesquet 2015). Hosokawa et al. (2001) reported that the addition of coniferyl alcohol to diluted zinnia culture medium with low cell density promoted the lignifications. They analysed the medium by GC-MS and HPLC and detected accumulation of the monolignols coniferyl aldehyde, coniferyl alcohol and synapyl alcohol. The levels of these substances were elevated at the beginning of SCWs thickening, then decreased during SCWs formation and increased again in the stage of lignification following the vacuole rupture. Based on these results it was suggested that monolignols are probably produced and secreted from parenchyma-like cells and from immature TEs. During TE differentiation and after cell death they are transported through the medium to be used in the lignification process of the differentiating living and dead TEs.

Dilignols are intermediates required for lignin polymerisation. Coniferyl alcohol, dilignols erythroguaiacylglycerol-β-coniferyl ether, threo-guaiacylglycerol-β-coniferyl ether, dehydrodiconiferyl alcohol and pinoresinol have been isolated from conditioned medium of differentiating zinnia cell culture (Tokunaga et al. 2005). Peaks of accumulation, especially for coniferyl alcohol were detected at the initial stage of transdifferentiation (indicating the substance is produced by the living cells) and in the final lignification stage (*post-mortem*) following TE autolysis. In the mid-term stage characterized with intensive SCW development, the concentration decreased indicating possible incorporation of coniferyl alcohol into developing SCWs. Mono- and dilignols are synthesised by enzymatic pathways involving PAL (Wang et al. 2013). The formation of TEs in a culture where PAL activity was inhibited by L-α-aminooxy-β-phenylpropionic acid was promoted at the addition of the above mentioned substances. This data showed that lignification indeed depends on the supply with mono- and dilignols which are produced and released into the medium from non-TEs and immature TEs. Although various models of translocation of monolignols are suggested, the molecular mechanism of their transport from the cytoplasm through the plasma membrane is not yet clear. As assumed by Novo-Uzal et al. (2013), the pathways may involve translocation through the 4-*O*-glucosylated forms, exocytosis via the ER-Golgi, passive diffusion via hydrophobic reactions through the plasma membrane or active transport via different transporters. In zinnia cell culture an implication of ATP binding cassette (ABC) transporters was assumed (Pesquet et al. 2005). In a recent work of Ménard and Pesquet (2015) it was suggested that *in planta* the intercellular interactions may contribute to the physiological functioning of xylem fibers. An example is the restoration from air embolisms, to which the lumen of xylem vessels is vulnerable. The authors proposed a mechanism of xylem sap flow recovery involving transportation of sugars and ions from xylem parenchyma cells into the TEs. This may increase the osmotic pressure of xylem sap which results in “flushing out” the air bubbles.

A contribution of zinnia Rac small guanosine-5'-triphosphatase **(**GTPase), ZeRAC2 to intercellular communication has been suggested by Nakanomyo et al*.* (2002). They found that *ZeRAC2* mRNA accumulates on the outer site of the plasma membrane of parenchyma cells and TE precursor cells both under xylogenesis inducing conditions *in vitro* and in zinnia plants. The localization of this protein was confirmed by confocal microscopy observations of the fusion protein GFP-ZeRAC2. Regarding the messenger function of ZeRAC2, it was speculated that as xylem parenchyma cells supply H_2_O_2_ necessary for the polymerization of lignin precursors, this protein might activate plasma membrane bound NADPH oxidase that generates the superoxide anion thus stimulating the further generation of H_2_O_2_ which is incorporated in the SCWs (Ogawa et al. 1997; Barceló 1998a, b). Hydrogen peroxide-dependent peroxidase and O^2-^ dependent laccases are involved in the production of lignin monomer radicals which are directly incorporated into the lignin in the cell wall (Ranocha et al. 1999; Boerjan et al. 2003; Barros et al. 2015; Ménard and Pesquet 2015; Serk et al. 2015). The extracellular transfer of H_2_O_2_ produced in non-differentiating cells and contributing to TEs *pre-* and *post-mortem* lignification was substantiated by inhibition of ROS generating enzymes. The inhibition resulted in suppression of TE lignification (Karlsson et al. 2005; Pesquet et al. 2013).

Extracellular Tracheary element Differentiation Inhibitory Factor (TDIF) – a peptide encoded by *CLAVATA3/ENDOSPERM SURROUNDING REGION (CLE)* genes has been found to repress the transdifferentiation in zinnia cell culture in the early stage (Ito et al. 2006). In *Arabidopsis* a ligand-receptor pair made of the TDIF peptide and TDIF RECEPTOR/ PHLOEM INTERCALATED WITH XYLEM membrane protein kinase (TDR/PXY) promotes proliferation of procambial cells and suppresses their xylem differentiation. It was suggested that from TDRF the extracellular signalling splits into two pathways independently contributing to sustaining the activity of pro-cambium and cambium stem cells and to suppressing the differentiation thus regulating the organization of vascular meristem *in planta*. At the intracellular level, it was shown that in the *Arabidopsis* xylem meristem, *CLE10* suppressed procambial cell differentiation through specifically repressing the expression of two *Arabidopsis Response Regulators* (*ARRs*), *ARR5* and *ARR6*, the products of which act as negative regulators of CK signalling (Kondo et al. 2011). Similar mechanism of control over TE differentiation may operate in zinnia cultured cells and during xylem differentiation in other plants (Fukuda et al. 2007; Hirakawa et al. 2010; Kondo et al. 2011).

Hydrolases released into the culture medium after vacuole rupture in differentiating TEs threaten the living cells. The reduced vitality of mesophyll cells may result in suppression of cell potential to transdifferentiate. However, it was established that the cells are capable of protecting themselves against the hydrolases by producing a specific peptide which is transported through the cell wall toward the extracellular space. The Tracheary Element Differentiation-related (TED4) peptide which is plant non-specific lipid transfer protein is demonstrated to perform a cell death protective function. The gene *ZeTED4* encoding for this peptide was found to be preferentially expressed in living and transdifferentiating cells. The gene was also expressed in association with vascular development of young zinnia seedlings (Endo et al. 2001). In xylogenic zinnia culture TED4 is secreted into the apoplast prior to and with the progression of morphological changes of the TEs. It was suggested that TED4 binds to and inactivates the proteasome in living mesophyll cells thereby inhibiting the proteasome mediated downstream cell death signalling (Endo et al. 2001; Escamez and Tuominen 2014). Further analysis will shed more light on the role of this and other PCD protective mechanisms in zinnia and other xylogenic systems.

**References**

Avci U, Petzold HE, Ismail IO, Beers EP, Haigler CH (2008) Cysteine proteases XCP1 and XCP2 aid micro-autolysis within the intact central vacuole during xylogenesis in *Arabidopsis* roots. Plant J 56:303-315. doi: 10.1111/j.1365-313x.2008.03592.x

Aoyagi S, Sugiyama M, Fukuda H (1998) *BEN1* and *ZEN1* cDNAs encoding S1-type DNases that are associated with programmed cell death in plants. FEBS Lett 429:134-138. [doi: 10.1016/S0014-5793(98)00563-8](http://dx.doi.org/10.1016/S0014-5793(98)00563-8)

[Barceló](http://link.springer.com/search?facet-author=%22A.+Ros+Barcel%C3%B3%22) AR (1998a) The generation of H_2_O_2_ in the xylem of Zinnia elegans is mediated by an NADPH-oxidase-like enzyme. Planta 207:207-216. doi: 10.1007/s004250050474

Barceló AR (1998b) Hydrogen peroxide production is a general property of the lignifying xylem from vascular plants. Ann Bot 82:97-103. doi: 10.1006/anbo.1998.0655

Barros J, Serk H, Granlund I, Pesquet E (2015) The cell biology of lignification in higher plants. Ann Bot 115:1053-1074. doi: 10.1093/aob/mcv046

Boerjan W, Ralph J, Baucher M (2003) Lignin biosynthesis. Annu Rev Plant Biol 54:519-546. doi: 10.1146/annurev.arplant.54.031902.134938

Bollhöner B, Prestele J, Tuominen H (2012) Xylem cell death: Emerging understanding of regulation and function. J Exp Bot 63:1081-1094. doi: 10.1093/jxb/err438

Bollhöner B, Zhang B, Stael S et al*.* (2013) *Post-mortem* function of AtMC9 in xylem vessel elements. New Phytol 200:498-510. doi: 10.1111/nph.12387

Dahiya P, Milioni D, Wells B, Stacey N, Roberts K, McCann MC (2005) A RING domain gene is expressed in different cell types of leaf trace, stem, and juvenile bundles in the stem vascular system of zinnia. Plant Physiol 138:1383-1395. doi: [​10.​1104/​pp.​104.​057901](http://dx.doi.org.ezproxy.library.wur.nl/10.1104/pp.104.057901)

# [Dahiya](http://link.springer.com.ezproxy.library.wur.nl/search?facet-author=%22Preeti+Dahiya%22) P, Findlay K, Roberts K, McCann MC (2006) A fasciclin-domain containing gene, ZeFLA11, is expressed exclusively in xylem elements that have reticulate wall thickenings in the stem vascular system of Zinnia elegans cv. Envy. Planta 223:1281-1291. doi: 10.1007/s00425-005-0177-9

Endo S, Demura T, Fukuda H (2001) Inhibition of proteasome activity by the TED4 protein in extracellular space: a novel mechanism for protection of living cells from injury caused by dying cells. Plant Cell Physiol 42:9-19. doi: 10.1093/pcp/pce002

Escamez S, Tuominen H (2014) Programmes of cell death and autolysis in tracheary elements: when a suicidal cell arranges its own corpse removal. J Exp Bot 65:1313-1321. doi: 10.1093/jxb/eru057

Farquharson KL (2014) Good neighbor hypothesis of lignification holds for xylem cells, but not for interfascicular fibers. Plant Cell 25:3635.  doi: <http:/​/​dx.​doi.​org/​10.​1105/​tpc.​113.​251010>

Fukuda J, Hirakawa Y, Sawa S (2007) Peptide signaling in vascular development. Curr Opin Plant Biol 10:477-482. doi 10.1016/j.pbi.2007.08.013

Groover A, DeWitt N, Heidel A, Jones A (1997) Programmed cell death of plant tracheary elements differentiating *in vitro*. Protoplasma 196:197-211. doi: 10.1007/BF01279568

# [Hirakawa Y](http://www.ncbi.nlm.nih.gov/pubmed/?term=Hirakawa%20Y%5BAuthor%5D&cauthor=true&cauthor_uid=20729381), [Kondo Y](http://www.ncbi.nlm.nih.gov/pubmed/?term=Kondo%20Y%5BAuthor%5D&cauthor=true&cauthor_uid=20729381), [Fukuda H](http://www.ncbi.nlm.nih.gov/pubmed/?term=Fukuda%20H%5BAuthor%5D&cauthor=true&cauthor_uid=20729381) (2010) TDIF peptide signaling regulates vascular stem cell proliferation via the WOX4 homeobox gene in *Arabidopsis*. Plant Cell 22:2618-2629. doi: 10.1105/tpc.110.076083

Hosokawa M, Suzuki S, Umezawa T, Sato Y (2001) Progress of lignification mediated by intercellular transportation of monolignols during tracheary element differentiation of isolated Zinnia mesophyll cells. Plant Cell Physiol 42:959-968. doi: 10.1093/pcp/pce124

Ito Y, Nakanomyo I, Motose H, Iwamoto K, Sawa S, Dohmae N, Fukuda H (2006) Dodeca-CLE peptides as suppressors of plant stem cell differentiation. Science 313:842-845. doi: 10.1126/science.1128436

# [Karlsson](http://jxb.oxfordjournals.org/search?author1=Marlene+Karlsson&sortspec=date&submit=Submit)[M, Melzer](http://jxb.oxfordjournals.org/content/56/418/2085.short#aff-1)M, Prokhorenko I (2005) Hydrogen peroxide and expression of hipI superoxide dismutase are associated with the development of secondary cell walls in Zinnia elegans. J Exp Bot 56:2085-2093. doi: 10.1093/jxb/eri207

Kondo Y, Hirakawa Y, Kieber JJ, Fukuda H (2011) CLE peptides can negatively regulate protoxylem vessel formation via cytokinin signaling. Plant Cell Physiol 52: 37-48. doi: 10.1093/pcp/pcq129

Ménard D, Pesquet E (2015) Cellular interactions during tracheary elements formation and function. Curr Opin Plant Biol 23:109-115. [doi:10.1016/j.pbi.2014.12.001](http://dx.doi.org/10.1016/j.pbi.2014.12.001)

Motose H, Fukuda H, Sugiyama M (2001a) Involvement of local intercellular communication in the differentiation of zinnia mesophyll cells into tracheary elements. Planta 213:121-131. doi: 10.1007/s004250000482

Motose H, Sugiyama M, Fukuda H (2001b) Cell-cell interactions during vascular development. J Plant Res 114:473-481. doi: 10.1007/pl00014014

Motose H, Sugiyama M, Fukuda H (2004) A proteoglycan mediates inductive interaction during vascular development. Nature 429:873-878. doi:10.1038/nature02613

Nakanomyo I, Kost B, Chua N-H, Fukuda H (2002) Preferential and asymmetrical accumulation of a Rac small GTPase mRNA in differentiating xylem cells of *Zinnia elegans*. Plant Cell Physiol 43:1484-1492. doi: 10.1093/pcp/pcf170

Novo-Uzal E, Fernбndez-Pйrez F, Herrero J, et al (2013) From *Zinnia* to *Arabidopsis*: approaching the involvement of peroxidases in lignification. J Exp Bot 64:3499- 3518. doi: 10.1093/jxb/ert221

Ogawa K, Kanematsu S, Asada K (1997) Generation of superoxide anion and localization of CuZn-superoxide dismutase in the vascular tissue of spinach hypocotyls: their association with lignification. Plant Cell Physiol 38:1118–1126.

Pesquet E, Ranocha P, Legay S, et al (2005) Novel markers of xylogenesis in zinnia are differentially regulated by auxin and cytokinin. Plant Physiol 139:1821-1839. doi: [​10.​1104/​pp.​105.​064337](http://dx.doi.org/10.1104/pp.105.064337)

Pesquet E, Zhang B, Gorzsas A, et al (2013) Non-cell-autonomous postmortem lignification of tracheary elements in Zinnia elegans. Plant Cell 25:1314-1328. doi: [10.1105/tpc.113.110593](http://dx.doi.org/10.1105%2Ftpc.113.110593)

Ranocha P, McDougall G, Hawkins S, et al (1999) Biochemical characterization, molecular cloning and expression of laccases - a divergent gene family - in poplar. Eur J Biochem 259:485-495. doi: 10.1046/j.1432-1327.1999.00061.x

# Sauter M (2015) Phytosulfokine peptide signaling. J Exp Bot 66:5161-5169. doi: 10.1093/jxb/erv071

Sehr EM, Agusti J, Lehner R, Farmer EE, Schwarz M, Greb T (2010) Analysis of secondary growth in the *Arabidopsis* shoot reveals a positive role of jasmonate signaling in cambium formation. Plant J 63:811-822. doi: 10.1111/j.1365-313x.2010.04283.x

Serk H, Gorzsás A, Tuominen H Pesquet E (2015) Cooperative lignification of xylem tracheary elements. Plant Signal Behav 10:4. e1003753. doi: 10.1080/15592324.2014.1003753.

Schuetz M, Smith R, Ellis B (2013) Xylem tissue specification, patterning, and differentiation mechanisms. J Exp Bot 64:11-31. doi: 10.1093/jxb/ers287

Tokunaga N, Sakakibara N, Umezawa T, Ito Y, Fukuda H, Sato Y (2005) Involvement of extracellular dilignols in lignification during tracheary element differentiation of isolated Zinnia mesophyll cells. Plant Cell Physiol 46:224-232. doi: 10.1093/pcp/pci017

Wang Y, Chantreau M, Sibout R, Hawkins S (2013) Plant cell wall lignification and monolignol metabolism. Front Plant Sci. doi: 10.3389/fpls.2013.00220

Yamamoto R, Fujioka S, Demura T, Takatsuto S, Yoshida S, Fukuda H (2001) Brassinosteroid levels increase drastically prior to morphogenesis of tracheary elements. Plant Physiol 125:556-563. doi: [​10.​1104/​pp.​125.​2.​556](http://dx.doi.org/10.1104/pp.125.2.556)

Yamamoto R. Fujioka S, Iwamoto K, et al (2007) Co-regulation of brassinosteroid biosynthesis-related genes during xylem cell differentiation. Plant Cell Physiol 48:74-83. doi: 10.1093/pcp/pcl039
