# Supplementary material for: Xylogenesis in zinnia (Zinnia elegans) cell cultures: unravelling the regulatory steps in a complex developmental programmed cell death event
Source: Planta. 2017 Feb 13;245(4):681–705. doi: 10.1007/s00425-017-2656-1 (PMC5357506; doi:10.1007/s00425-017-2656-1)
Supplement: Supplementary file 6 — Supplementary material 6 (DOCX 21 kb) [file 425_2017_2656_MOESM6_ESM.docx]

Title: **Xylogenesis in zinnia (*Zinnia elegans*) cell cultures: unravelling the regulatory steps in a complex developmental programmed cell death event**

Journal: Planta

Authors: Elena T. Iakimova^1^, Ernst J. Woltering^2,3^

^1^Institute of Ornamental Plants, Sofia, Bulgaria, ^2^Wageningen University and Research, Food & Biobased Research and ^3^Wageningen University, Horticulture and Product Physiology, Wageningen, The Netherlands

Corresponding author: Ernst J. Woltering

e-mail: ernst.woltering@wur.nl

**Examples of approaches to manipulate TE dimensions**

It was established that TE dimensions of *in vitro* differentiating zinnia cells can be manipulated by chemical treatments applied to culture medium and by modifications of culture conditions. The delay of the timing of TE cell death execution following treatments with inhibitors of CLPs and cell death-associated cysteine proteases can result in enlarged TE dimensions (Twumasi et al. 2010a). Evidence that TE size can be controlled by exogenous substances comes also from the experiments of Muñiz et al. (2008). They reported that supplementing the culture with the PA spermine retarded the TE formation in a concentration dependent manner and caused over threefold increase of the width and twofold increase of the length of the TEs. The effect of spermine on TE dimensions was assumed to be due to involvement of this PA in the regulation of TE cell death (Muñiz et al*.* 2008). These findings show that the progress of transdifferentiation and the size of TEs in zinnia culture can be manipulated by exogenous agents affecting the PCD signal transduction cascade.

Lee and Roberts (2004) demonstrated that fluctuations of the extracellular osmolarity in zinnia culture may influence the TE differentiation through affecting the expansion of mesophyll cells in the stage before hormonal induction. At lower extracellular or cellular osmotic pressure the cell expansion was stimulated and differentiation was prevented and *vice verse*. This suggested that in the zinnia cell system and possibly *in planta* the transition from non-differentiation to differentiation activity might be dependent on the osmotic equilibrium established across the cell membrane between intra- and intercellular environment. The role of osmolarity has been supported by the studies of Twumasi et al. (2010b) who examined the effect of light intensity (LI) and electrical conductivity (EC) during the growth of zinnia seedlings of two cultivars on the initial leaf osmolarity (LO), on the dimensions of mesophyll cells in intact leaves and, on the rate of differentiation and TE dimensions in the xylogenic culture. For experiments related to LI, the plants were grown on commercial peat-based substrate under different LI and for the trial related to EC the plants were grown in liquid growth medium with EC set at different levels. The authors established that the LO during growth varied between 300 and 400 mOsm and was positively correlated with the increases of LI and EC. The mesophyll cell size was reduced at higher EC and enlarged at higher LI. Further, in *in vitro* cultured mesophyll cells, it was observed that the higher leaf osmotic potential correlated with increased frequency of TE formation and enhanced size of differentiated TE. Cultivar differences in the transdifferentiation capacity of isolated cells were detected. The authors concluded that the appropriate light quality and the optimisation of EC in root environment during growing period may result in enhancement of TE differentiation in the cell culture. In other studies light and hormone dependent expansion of mesophyll cells related to the timing of occurrence and the size of formed TEs in zinnia cell culture was also shown (Lee et al. 2000).

**References**

Lee S, Woffenden BJ, Beers EP, Roberts AW (2000) Expansion of cultured mesophyll cells in response to hormones and light. Physiol Plantarum 108:216-222. doi: 10.1111/j.1399-3054.2011.01538.x

Lee S, Roberts AW (2004) Tracheary element differentiation is correlated with inhibition of cell expansion in xylogenic mesophyll suspension cultures. Plant Physiol Bioch 42:43-48. [doi:10.1016/j.plaphy.2003.10.05](http://dx.doi.org/10.1016/j.plaphy.2003.10.005)

Muñiz L, Minguet EG, Singh SK*,* et al (2008) ACAULIS5 controls *Arabidopsis* xylem specification through the prevention of premature cell death. Development 135:2573-2582. doi: 10.1242/dev.019349

Twumasi P, Iakimova ET, Qian D. et al (2010a) Delayed programmed cell death affects the kinetics and dimensions of tracheary elements in xylogenic zinnia *(Zinnia elegans)* cells. BMC Plant Biol 10:162. doi: 10.1186/1471-2229-10-162

Twumasi P, Schel J, van Ieperen W (2010b) Osmotic potential of *Zinnia elegans* plant material affects the yield and morphology of tracheary elements produced *in vitro*. African J Biotechnol 9:8712-8721. doi: 10.5897/ajb10.1150
